# Screening the γ-core Motif Peptides of Ascomycetous Antifungal Proteins for Antifungal Activity and Potential Therapeutic Applicability

**DOI:** 10.1007/s12602-025-10890-y

**Published:** 2026-01-06

**Authors:** John K Karemera, Györgyi Váradi, Gábor Bende, Richárd Merber, Kinga Dán, Csaba Papp, Attila Farkas, Gergely Maróti, Gábor K. Tóth, Attila Borics, László Galgóczy

**Affiliations:** 1 Department of Biotechnology and Microbiology, Faculty of Science and Informatics, University of Szeged, Szeged, Hungary; 2 Doctoral School of Biology, Faculty of Science and Informatics, University of Szeged, Szeged, Hungary; 3 Department of Medical Chemistry, Albert Szent-Györgyi Medical School, University of Szeged, Szeged, Hungary; 4 Department of Theoretical Health Sciences and Health Management, Faculty of Health Sciences and Social Studies, University of Szeged, Szeged, Hungary; 5 Institute of Plant Biology, HUN-REN Biological Research Centre, Szeged, Hungary; 6 Institute of Biochemistry, HUN-REN Biological Research Centre, Szeged, Hungary

**Keywords:** Antifungal peptide, Γγ-core motif, Peptide-drug interaction, Therapeutic efficacy, *Candida albicans*, *Aspergillus fumigatus*

## Abstract

The incidence of fungal infections has increased markedly in recent years, driven by increasing resistance to conventional antifungal agents. To address this challenge, the World Health Organization has highlighted the urgent need for novel antifungal molecules, particularly those that can enhance the efficacy of existing drugs. Synthetic peptides (γAFPs), derived from conserved γ-core motifs (GXC-X_3 − 9_-C) of antifungal proteins, represent a potential source of such combination partners. Here, we systematically screened 19 γAFPs of various fungal origins and physicochemical properties to assess their activity and interaction with conventional agents. The intrinsic antifungal activity of these peptides was primarily governed by their net positive charge and hydrophilicity, with the charge-to-hydropathy ratio emerging as a strong predictor of efficacy. From this panel, two peptides (γAFP^B6GXZ8^ from *Penicillium rubens* and γAFP^A0A2J5HZT4^ from *Aspergillus taichungensis*) were identified as the leading candidates with potent antifungal activity mediated by the disruption of the plasma membrane. Although their standalone antifungal potency was modest, both peptides exhibited robust synergistic interactions with clinically used antifungal agents in vitro: γAFP^B6GXZ8^ enhanced terbinafine efficacy against *Candida albicans*, while γAFP^A0A2J5HZT4^ potentiated fluconazole activity against *Aspergillus fumigatus*. In a *Galleria mellonella* infection model, neither the peptides nor their combinations with conventional antifungal agents caused host toxicity, and effectively prevented *C. albicans* infection and prolonged the survival of larvae infected with *A. fumigatus*. These findings confirm that the synergistic effects observed in vitro can be maintained in vivo. Collectively, our findings identify two γ-core-derived peptides as well-tolerated synergistic co-therapeutics that augment antifungal drug efficacy and constitute promising templates for directed optimization.

**Supplementary Information:**

The online version contains supplementary material available at 10.1007/s12602-025-10890-y.

## Introduction

Fungal infections, ranging from superficial skin conditions to life-threatening systemic diseases, represent an increasing global health concern that affect billions of people globally and contribute to substantial morbidity and mortality each year [1]. Recent studies reported annual fungal-related death estimates ranging from 1.7 million [2] to 3.8 million [3]. Several factors contribute to this phenomenon, including the limited availability of antifungal drugs, rising antifungal resistance, and the lack of novel antifungal agents with different mechanisms of action [4]. Considering these challenges, the World Health Organization advocates for a coordinated effort among researchers, healthcare providers, and policymakers to develop new antifungal therapies and optimize existing treatment protocols [5].

Antimicrobial peptides and proteins with antifungal properties (AFPs) have emerged as promising therapeutic compounds for the treatment of fungal infections [6, 7]. These peptides disrupt fungal cell membranes and interfere with intracellular processes, rendering them effective against a broad spectrum of fungal pathogens, including drug-resistant strains. AFPs can enhance the efficacy of conventional antifungal drugs and mitigate the risk of resistance. Furthermore, rational design strategies that incorporate amino acid substitutions have the potential to improve the stability and efficacy of AFPs while reducing toxicity, increasing thus their suitability for therapeutic applications [8]. Given these advancements, several AFPs are currently undergoing clinical trials to assess their safety and effectiveness in the treatment of fungal infections [4].

The γ-core motif (GXC-X_3 − 6_-C signature, where X represents any amino acid) is a conserved structural element found in numerous AFPs in both plants and animals that contributes significantly to their antimicrobial properties. These γ-core motif-containing AFPs can disrupt intracellular processes such as potassium ion homeostasis and mitochondrial function, leading to elevated intracellular ATP levels and eventual cell death [9]. Rationally designed synthetic short peptides encompassing the γ-core motif of plant AFPs exhibit broad-spectrum antifungal activity against various fungi, positioning them as promising candidates for the development of novel antifungal agents [10, 11]. Depending on the amino acid composition and the resulting physicochemical properties, peptides rationally designed on the basis of the γ-core motifs of plant AFPs induce hyperbranching in hyphae, thus inhibiting fungal growth, or disrupt fungal membranes, leading to cell lysis [11, 12].

The γ-core motif is evolutionarily conserved across various classes of antifungal proteins in filamentous Ascomycetes, particularly within the class Eurotiomycetes [13]. This conservation suggests that the γ-core motif plays a crucial structural and/or functional role in these proteins. To support this hypothesis, previous research demonstrated that the antifungal efficacy and overall tertiary structure of AFPs from *Penicillium chrysogenum* [13], *Penicillium expansum* [14], and *Aspergillus fischeri* (formerly *Neosartorya fischeri*) [15] can be modulated by amino acid substitutions within the γ-core motif, which is localized in a loop region between two β-strands. However, peptides synthesized on the basis of the native γ-core motif of *P. chrysogenum* PAF and *A*. *fischeri* NFAP and NFAP2 exhibit weak or no growth inhibitory activity against fungi [13, 16–18]. By rationally modifying specific amino acids within the native γ-core motif, synthetic peptide variants with significantly enhanced antifungal properties can be developed to target a wide range of human and plant pathogenic fungi [13, 16–19].

This study investigated the antifungal efficacy and therapeutic applicability of synthetic peptides that encompass the γ-core motifs of all Eurotiomycetes antifungal protein clades (γAFPs). Unlike previous work [13, 16–19], here we systematically selected γ-core motifs of AFPs representing four phylogenetically distinct groups within Eurotiomycetes [13], allowing the design of γAFP peptides with various physicochemical properties. We demonstrated that the in vitro antifungal effectiveness of these peptides was strongly influenced by the balance between net charge and the grand average of hydropathy (GRAVY). Importantly, this study is the first (to the best of our knowledge) to investigate interactions between antifungal-active γAFPs and conventional antifungal agents, revealing synergistic effects that led to enhanced efficacy against human pathogenic yeast and filamentous fungal isolates under both in vitro and in vivo conditions. These findings support the clinical potential of γAFPs and their combinations with conventional antifungal drugs in the treatment of fungal infections.

## Materials and Methods

### *In Silico* Analyses

The amino acid sequences of 15 AFPs from various Eurotiomycetes species were retrieved from the UniProt database [20]. Sequence alignment was performed using BioEdit [21] and visualized using Jalview version 2 [22]. The signal sequence cleavage sites were predicted using SignalP 5.0 [23].

A maximum likelihood phylogenetic tree was constructed using MEGA11 by applying the WAG substitution model, gamma-distributed rate variation, and the nearest-neighbor interchange algorithm, with 1,000 bootstrap replicates [24, 25]. Defensin-like proteins of *Raphanus sativus* (UniProt IDs: P69241, P30230) served as an outgroup.

The physicochemical properties of γAFPs were determined using the Expasy ProtParam tool [26] and the Antimicrobial Peptide Calculator and Predictor available from the Antimicrobial Peptide Database [27]. The tertiary structures of AFPs were obtained from AlphaFold through UniProt [28, 29], while those of γAFPs were predicted using PEP-FOLD3 [30].

All tertiary structures were visualized using the UCSF Chimera [31]. Model reliability was assessed using Ramachandran plots generated via MolProbity [32].

### Peptide Synthesis

The γAFPs were produced through microwave-assisted, stepwise solid-phase peptide synthesis employing Fmoc/S-tBu chemistry on a Liberty Blue synthesizer (CEM Corporation, Matthews, NC, USA). Synthesis was carried out using TentaGel S RAM resin (loading: 0.2 mmol/g) with coupling facilitated by ethyl 2-cyano-2-(hydroxyimino)acetate and diisopropylcarbodiimide. The peptides were cleaved from the resin using a mixture containing trifluoroacetic acid (TFA), water, and dithiothreitol at a ratio of 95:5:3 (v/v/v) and incubated for 3 h. Post-cleavage, TFA was evaporated, and the peptides were precipitated using ice-cold diethyl ether, subsequently dissolved in 10% acetic acid (v/v), and freeze-dried. The resulting crude peptides were purified by semipreparative reverse-phase high-performance liquid chromatography (RP–HPLC) using a solvent system of 0.1% TFA (v/v) as solvent A and 80% acetonitrile containing 0.1% TFA (v/v) as solvent B. A linear gradient from 0% to 30% solvent B over 60 min was applied. Purification was conducted on a Phenomenex Jupiter Proteo 90 Å column (250 mm × 10 mm) with a Shimadzu HPLC system (Shimadzu, Tokyo, Japan), and the absorbance was monitored at 220 nm. The purity of the peptides was assessed by analytical RP–HPLC on a Phenomenex Luna 10 μm C18 100 Å column operated with an Agilent 1100 HPLC system (Agilent, Palo Alto, CA, USA). All γAFPs were obtained at ≥ 97% purity, as determined by analytical RP–HPLC. Lyophilized γAFPs were stored at − 20 °C pending further application.

### Fungal Strains and Inoculum Preparation

Fresh conidial suspensions of molds (*Aspergillus fumigatus* CBS 101355, *Botrytis cinerea* SZMC 21472, *Cladosporium herbarum* FSU 1148, *Fusarium subglutinans* CBS 747.97) and cells of mid-log phase yeast cultures (*Candida albicans* SC5314, *Saccharomyces cerevisiae* SZMC 0644) were used for all experiments. The yeasts were maintained on yeast extract peptone dextrose (YPD) agar slants [1% yeast extract, 2% peptone, 2% glucose, 2% agar (w/v)], whereas molds were grown on potato dextrose agar (PDA, Sigma-Aldrich, St. Louis, MO, USA) at 4 °C. Fresh conidia were harvested from the surface of 7-day-old mold cultures grown on PDA at 25–30 °C (*A. fumigatus*), suspended in spore buffer [0.9% NaCl, 0.01% Tween (v/v)], and filtered using a 40-µm pore-size cell strainer (VWR, Radnor, PA, USA). The conidia were subsequently washed twice in spore buffer (900 × *g* for 5 min) and resuspended in spore buffer. To generate mid-log phase yeast cultures, cells were inoculated from YPD agar slants into low cationic medium [LCM; 0.5% glucose, 0.25% yeast extract, 0.0125% peptone (w/v)] and incubated at 30 °C for 8 h with continuous shaking (200 rpm). The cultures were then inoculated at a 1:100 dilution into fresh LCM and further incubated under the same conditions for 16 h. Finally, both conidia and yeast cells were diluted in LCM to achieve the concentrations required for the experiments.

### *In Vitro* Antifungal Susceptibility Tests

A broth microdilution susceptibility assay was performed as described by Tóth et al. (2016) [33] to evaluate the antifungal efficacy of synthetic γAFPs against four molds (*A*. *fumigatus* CBS 101355, *B. cinerea* SZMC 21472, *C. herbarum* FSU 1148, *F. subglutinans* CBS 747.97) and two yeasts (*C. albicans* SC5314, *S. cerevisiae* SZMC 0644) strains in LCM. Briefly, 100 µL of γAFPs (25–400 µg/mL, 2-fold serial dilutions in LCM) were combined with 100 µL of fungal suspensions (2 × 10^5^ conidia or yeast cells/mL) in flat-bottom 96-well microtiter plates (TC Plate 96 Well, Suspension, F; Sarstedt, Nümbrecht, Germany). LCM lacking γAFP served as the untreated control. The plates were statically incubated at 25 °C for 72 h (*B. cinerea* SZMC 21472, *C. herbarum* FSU 1148, *F. subglutinans* CBS 747.97) or at 30 °C for 48 (*C. albicans* SC5314, *S. cerevisiae* SZMC 0644) or 72 h (*A. fumigatus* CBS 101355). The absorbance at 620 nm (OD_620_) was recorded using a SPECTROstar Nanoplate reader (BMG Labtech, Ortenberg, Germany). Fresh LCM (200 µL) was used for background calibration. The minimum inhibitory concentration (MIC) of γAFPs was defined as the lowest concentration that reduced fungal growth to ≤ 5% relative to the untreated control level (OD_620_ set to 100%). In the cases in which no MIC was achieved, the growth inhibition percentage (IP – the percentage reduction in fungal growth relative to the untreated control in the broth microdilution assay) at 200 µg/mL γAFP was calculated as follows: IP = 100%−[(absorbance of treated culture × 100)/absorbance of untreated culture]. For this calculation, the absorbance of the untreated culture was set to 100%, and fresh medium (200 µL) was used for spectrophotometric calibration. Susceptibility tests were carried out at least twice, each including two technical replicates.

The broth microdilution method, as previously described, was applied to determine the MICs of conventional antifungal drugs (all from MedChemExpress, Monmouth Junction, NJ, USA), including amphotericin B (AMB), fluconazole (FLC), micafungin (MFG), and terbinafine (TRB), against *A. fumigatus* CBS 101355 and *C. albican*s SC5314. The tested concentration ranges were 0.125–32, 2–32, 0.078–32, and 0.125–32 µg/mL for AMB, FLC, MFG, and TRB, respectively.

### *In Vitro* Interactions between γAFPs and Antifungal Drugs

The checkerboard titration method [34] was employed to investigate the interactions between γAFPs and antifungal drugs against *C. albicans* SC5314 and *A. fumigatus* CBS 101355. In this experiment, 100 µL of 2-fold serial dilutions of γAFP (ranging from 4 × MIC, prepared in 10 steps in LCM) were combined with 100 µL of 2-fold serial dilutions of the antifungal drugs (4 × MIC, prepared in 10 steps in LCM containing fungal cells or conidia at 2 × 10^5^ per mL). The plates were statically incubated at 30 °C for 48 h (*C. albicans* SC5314) or at 25 °C for 72 h (*A. fumigatus* CBS 101355). After incubation, the percentage of growth in the presence of γAFP was calculated according to OD_620_ using the formula: percent growth = (absorbance of treated culture × 100)/absorbance of untreated culture. For this calculation, the absorbance of the untreated culture was set to 100%, and fresh medium (200 µL) was used for spectrophotometric calibration. The interaction ratio (IR – the quantitative assessment of whether two agents interact synergistically, additively, or antagonistically) was determined using the Abbott formula [35] (Moreno et al., 2003) as follows: IR = I_o_/I_e_, where I_e_ = X + Y−(XY/100) (expected percentage inhibition for a given interaction), X and Y represent the percent inhibition of the individual compounds used alone, and I_o_ is the observed percent inhibition. The nature of the interaction was considered additive if IR ranged 0.5–1.5, synergistic if IR > 1.5, and antagonistic if IR < 0.5. There was no interaction when the combined effect was similar to that of the stronger individual compound. The interaction experiments were repeated twice, each including two technical replicates.

### Electronic Circular Dichroism (ECD) Spectroscopy

The secondary structural features of γAFPs were characterized by ECD spectroscopy. Spectral data were recorded between 195 and 260 nm using a Jasco-J815 spectropolarimeter (JASCO, Tokyo, Japan). Prior to measurement, peptide solutions were prepared at a concentration of 100 µg/mL in double-distilled water (ddH_2_O) and placed in quartz cuvettes with a 0.1-cm optical path length. The temperature was maintained at 25 °C throughout the acquisition process using a Peltier-based thermoelectric unit (TE Technology, Traverse City, MI, USA). Each spectrum represents an average of 10 consecutive scans per sample, with the solvent spectra subtracted to isolate the peptide-specific signals. Ellipticity was expressed in mean residue molar ellipticity units. Estimations of the secondary structural content, reflecting canonical motifs, were performed via the DichroWeb server [36] using the CDSSTR analytical method [37].

The conformational changes in the secondary structures of γAFP^B6GXZ8^ and γAFP^A0A2J5HZT4^ in the presence of *C. albicans* SC5314 cells, *A. fumigatus* CBS 101355 conidia, TRB, FLC, and their respective synergistic combinations (TRB + γAFP^B6GXZ8^ – *C. albicans* SC5314 cells and FLC + γAFP^A0A2J5HZT4^ – *A. fumigatus* CBS 101355 conidia) were investigated using ECD spectroscopy following the measurement conditions previously described. For this analysis, conidia or yeast cells were washed three times and resuspended in ddH_2_O or an aqueous solution of γAFP^B6GXZ8^ (200 µg/mL), γAFP^A0A2J5HZT4^ (200 µg/mL), TRB (1 µg/mL) or FLC (32 µg/mL), and γAFP^B6GXZ8^ (200 µg/mL) + TRB (0.5 µg/mL) or γAFP^A0A2J5HZT4^ (200 µg/mL) + FLC (32 µg/mL) at a final concentration of 2 × 10^7^ cells or conidia/mL. The spectra of ddH_2_O, aqueous solutions of γAFPs, antifungal drugs, and their combinations were also acquired for background subtraction.

### Fluorescence-activated Cell Sorting (FACS)

The antifungal efficacies of γAFP^B6GXZ8^, γAFP^A0A2J5HZT4^, TRB, FLC, and their respective synergistic combinations (γAFP^B6GXZ8^ + TRB, γAFP^A0A2J5HZT4^ + FLC) were evaluated by FACS. *C. albicans* SC5314 cells and *A. fumigatus* CBS 101355 conidia (2 × 10^5^) were treated with 200 µg/mL γAFP^A0A2J5HZT4^, 200 µg/mL γAFP^B6GXZ8^, 32 µg/mL FLC, 1 µg/mL TRB, and combinations namely 200 µg/mL γAFP^A0A2J5HZT4^ + 32 µg/mL FLC and 200 µg/mL γAFP^B6GXZ8^ + 0.5 µg/mL TRB, in LCM at 30 °C for 1 h with shaking (160 rpm). The treated cells were then stained with 5 µg/mL propidium iodide (PI; Sigma-Aldrich) for 10 min at room temperature in the dark, washed twice in PBS (9000 × *g* for 5 min), and resuspended in PBS. For positive PI staining and calibration controls, cells or conidia were treated with 70% ethanol (v/v) for 10 min at room temperature under shaking conditions (160 rpm). Untreated cells or conidia functioned as controls to reflect spontaneous cell death. PI-positive signals were detected using a FlowSight imaging flow cytometer (Amnis^®^, MilliporeSigma, Burlington, MA, USA) equipped with excitation lasers at wavelengths of 405 (violet), 488 (blue), and 642 nm (red). Calibration procedures were included to avoid fluorescence channel oversaturation and prevent artifact signals attributable to spectral spillover. Each experimental run analyzed a total of 5000 cells. PI-associated fluorescence was detected at 642 nm, with excitation lasers and emission in the channel 2 window. Gating strategies were refined to include at least 96% of the untreated population while excluding cellular debris from data collection. Flow cytometry datasets were processed and interpreted using Amnis Image Data Exploration and Analysis Software (MilliporeSigma). All FACS experiments were repeated three times independently.

### Scanning Electron Microscopy (SEM)

SEM was performed to examine the morphological effects of γAFP^B6GXZ8^ (200 µg/mL), γAFP^A0A2J5HZT4^ (200 µg/mL), TRB (1 µg/mL), FLC (32 µg/mL), and their respective synergistic combinations [γAFP^B6GXZ8^ (200 µg/mL) + TRB (0.5 µg/mL), γAFP^A0A2J5HZT4^ (200 µg/mL) + FLC (32 µg/mL)] on *C. albicans* SC5314 cells and *A. fumigatus* CBS 101355 conidia (4 × 10^6^ cells or conidia) under the following conditions: LCM, incubation at 30 °C for 16 h with shaking at 160 rpm for *C. albicans*, and under static conditions for *A. fumigatus*. Untreated conidia or cells served as morphological controls. Cells or conidia were harvested (9000 × *g* for 5 min), washed twice, and resuspended in PBS. For SEM, 8-µL aliquots were dispensed onto silicon disks precoated with 0.01% (w/v) poly-l-lysine (Merck Millipore, Billerica, MA, USA). Samples were fixed overnight at 4 °C in 2.5% (v/v) glutaraldehyde with 0.05 M cacodylate buffer (pH 7.2) in PBS. After fixation, the specimens were rinsed twice with PBS and dehydrated through a graded ethanol series (30%–100%, v/v; 4 h per concentration at 4 °C). The samples were dried using a Quorum K850 critical-point dryer (Quorum Technologies, Laughton, UK), coated with a 12 nm gold layer, and imaged using a JEOL JSM-7100 F/LV field emission SEM (JEOL Ltd., Tokyo, Japan).

### Hemolysis Assay

The hemolytic activities of aqueous solutions containing γAFP^B6GXZ8^ (200 µg/mL), γAFP^A0A2J5HZT4^ (200 µg/mL), TRB (1 µg/mL), FLC (32 µg/mL), and their respective synergistic combinations [γAFP^B6GXZ8^ (200 µg/mL) + TRB (0.5 µg/mL), γAFP^A0A2J5HZT4^ (200 µg/mL) + FLC (32 µg/mL)] were assessed using Columbia blood agar plates (5% (v/v) sheep blood; VWR, Radnor, PA, USA). Sterile filter paper disks (6 mm diameter) were impregnated with 10 µL of each solution and placed onto agar plates. Sterile ddH_2_O and 20% (v/v) Triton X-100 were used as negative and positive hemolysis controls, respectively. The presence of clear zones surrounding the filter disks was examined after 24 h of incubation at 37 °C. The experiment was carried out in triplicate.

### *Galleria Mellonella* Toxicity Assay

The potential in vivo toxic effects of γAFP^B6GXZ8^, γAFP^A0A2J5HZT4^, TRB, FLC, and their respective synergistic combinations (γAFP^B6GXZ8^ + TRB, γAFP^A0A2J5HZT4^ + FLC) were investigated in *G. mellonella* larvae at the concentrations used in the hemolysis assay. Twenty microliters of each test solution, prepared in insect physiological saline (IPS; 50 mM NaCl, 5 mM KCl, 10 mM EDTA, and 30 mM sodium citrate in 0.1 M Tris-HCl, pH 6.9), were injected intrahemocoelically using 29-gauge insulin needles (BD Micro-Fine, Franklin Lakes, NJ, USA) through the last right prolegs of 20 larvae. The larvae were incubated at 37 °C, and survival was monitored every 24 h for 6 days. IPS- and 20% (v/v) Triton X-100–treated larvae served as nontoxic and positive toxicity controls, respectively, whereas larvae without any interventions served as untreated controls. The toxicity assay was repeated twice.

### *In Vivo* Therapeutic Efficacy of γAFP + Antifungal Drug Combinations

The in vivo therapeutic efficacies of γAFP^B6GXZ8^, γAFP^A0A2J5HZT4^, TRB, FLC, and their respective combinations (γAFP^B6GXZ8^ + TRB, γAFP^A0A2J5HZT4^ + FLC) used in the hemolysis assay were evaluated in *G. mellonella* larvae following the protocol of the toxicity assay. In addition to the procedure described above, 20 µL of fungal cell or conidial suspension (2 × 10^7^ conidia or cells/mL) were injected into the last left prolegs of larvae, whereas in the case of the nontoxic and positive toxicity controls, 20 µL of IPS was injected. This experiment was repeated twice.

### Statistical Analysis

For growth inhibitory activity, one-way ANOVA and Tukey’s HSD post-hoc test (Statistics Kingdom, https://www.statskingdom.com/index.html) were applied to determine significant differences (*p* ≤ 0.05) regarding the proportion of dead cells following various treatments (Statistics Kingdom, 2022). To evaluate statistically significant differences in FACS results, Pearson’s chi-squared test was applied, and the phi coefficient was calculated to evaluate the strength of association between two treatment groups (Statistics Kingdom online platform, 2025; https://www.statskingdom.com/310GoodnessChi.html). To assess *G. mellonella* survival, log-rank (Mantel–Cox) and Gehan–Breslow–Wilcoxon tests were performed using GraphPad Prism 7.00 (GraphPad Software, Boston, MA, USA). Survival differences were deemed statistically significant at *p* ≤ 0.05 in both tests. All statistical analyses were conducted using GraphPad Prism 7.00 (GraphPad Software, Boston, MA, USA).

Figure S1 presents a flow chart outlining the study design.

## Results

### AFP Selection for Peptide Design and Physicochemical Properties of γAFPs

**Fig. 1:**
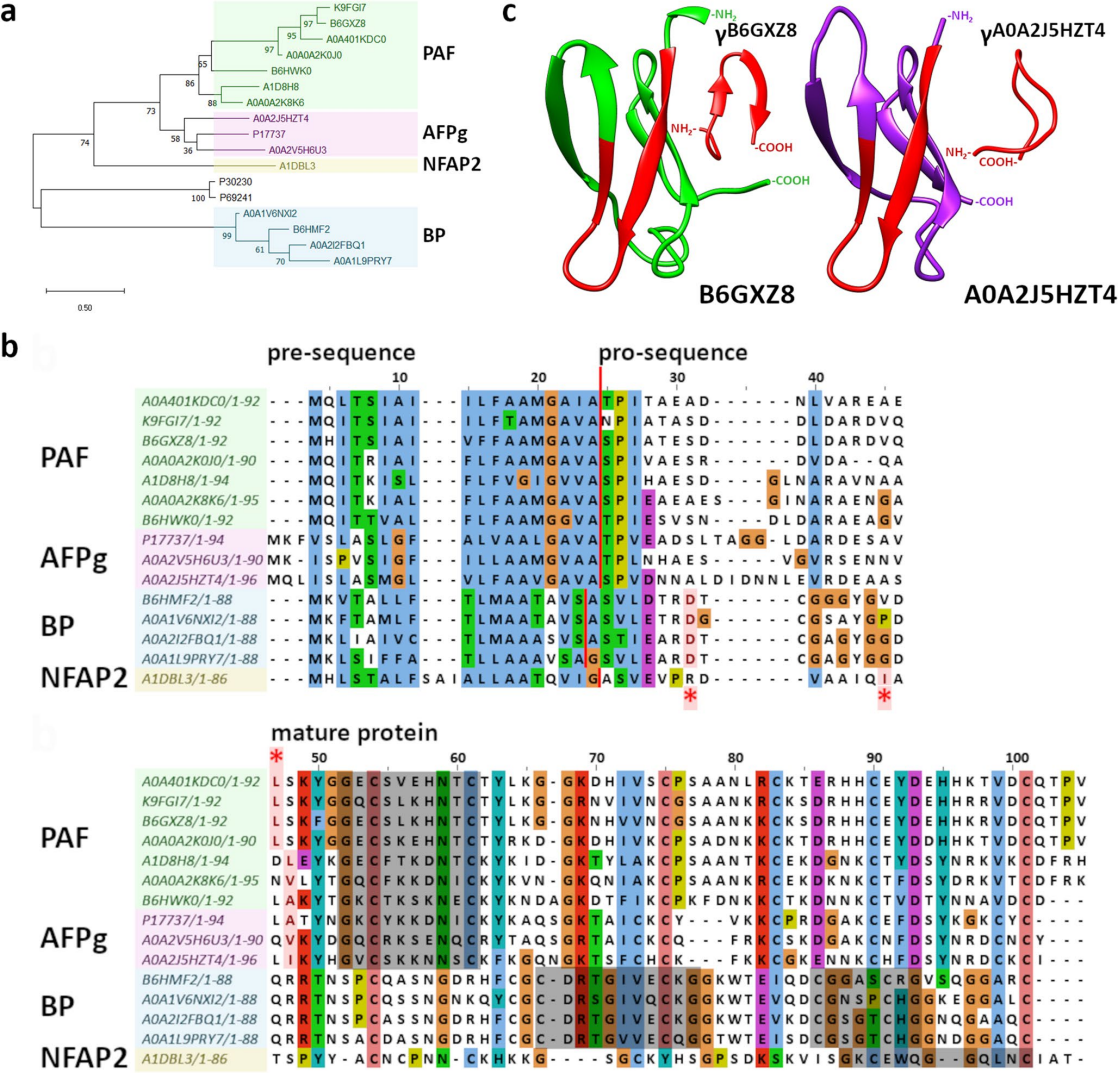
Phylogenetic and in silico structural analyses of antifungal proteins from Eurotiomycetes involved in this study. Maximum likelihood tree (**a**), and ClustalW multiple alignment of AMPs (**b**), whereon the UniProt database accession numbers of the respective AFPs are indicated (For further information see Table 1). The *Penicillium chrysogenum* antifungal protein (PAF), *Aspergillus giganteus* antifungal protein (AFPg), *Neosartorya* (*Aspergillus*) *fischeri* antifungal protein 2 (NFAP2), and the *Penicillium brevicompactum* ‘bubble’ protein (BP) subclades are highlighted in green, purple, yellow, and blue, respectively. In panel (**b**), red line indicates the predicted cleavage site of the signal sequence, and the first amino acid of the mature AFP is highlighted in red and indicated with red asterisk, the conserved γ-core motif (GXC-X_3 − 9_-C) is highlighted in grey. AlphaFold and PEP-FOLD3 predicted tertiary structure of *Penicillium rubens* Wisconsin 54–1255 PAF-like (B6GXZ8), and *Aspergillus taichungensis* IBT 19,404 AFP-like (A0A2J5HZT4) proteins, and the synthetic peptides designed on the γ-core motifs (γ^B6GXZ8^ and γ^A0A2J5HZT4^) (**c**)

Previous studies demonstrated that net charge and hydrophobicity significantly influence the antifungal efficacy of synthetic peptides designed using the γ-core motif of AFPs of *P. chrysogenum* and *A*. *fischeri* [13, 16–19]. Considering these findings, γ-core motifs of representative AFPs belonging to four phylogenetically distinct groups within Eurotiomycetes (Figs. 1a and b, Table S1) were selected for γAFP design based on their differences in physicochemical properties (Table 1). To optimize their antifungal activity, the designed γAFPs incorporated three additional amino acids from the N-terminus and one additional amino acid from the C-terminus as described by Sonderegger et al. (2018) [13] (Table 1). The γAFPs encompassing the γ-core motifs of the *P. chrysogenum* PAF group exhibited net charge variations between − 1.5 and + 4.0, with GRAVY ranging from − 1.814 to − 0.607. For γAFPs derived from the *Aspergillus giganteus* AFP group, the net charge ranged between + 2.0 and + 4.25, with GRAVY ranging from − 2.271 to − 1.421. For γAFPs from the *Penicillium brevicompactum* “bubble” protein (BP) group, net charge varied between − 0.75 and + 2.0, whereas GRAVY ranged from − 1.350 to − 0.275. Finally, the γAFP of the *A*. *fischeri* NFAP2 group exhibited a neutral charge and an almost zero GRAVY value (0.075) (Table 1). The elevated Boman index (> 2.50) observed in certain members of the PAF (i.e., γAFP^A0A0A2K0J0^, γAFP^A1D8H8^, γAFP^B6HWK0^), AFPg (i.e., γAFP^P17737^, γAFP^A0A2V5H6U3^, γAFP^A0A2J5HZT4^), and BP groups (i.e., γAFP2^A0A1V6NXI2^) suggests high binding potential to membranes, supporting the hypothesis that they interact with the cell membrane [38].

**Table 1:** Physicochemical properties of synthetic peptides spanning the γ-core motif (γAFP) of AFPs from eurotiomycetes

Peptide	Length (aa)	Mw (Da)	Theoretical pI	Net charge (pH = 7.0)	GRAVY	Boman index (kcal/mol)
PAF-group						
*Aspergillus awamori* (A0A401KDC0): KYGGECSVEHNTCT						
γAFP^A0A401KDC0^	14	1527.64	5.40	−0.75	−0.907	2.19
*Penicillium digitatum* (K9FGI7): KYGGQCSLKHNTCT						
γAFP^K9FGI7^	14	1539.74	8.86	+ 2.5	−0.964	1.94
*Penicillium rubens* (B6GXZ8): KFGGECSLKHNTCT						
γAFP^B6GXZ8^*^#^	14	1524.73	8.06	+ 1.25	−0.671	1.81
*Penicillium expansum* (A0A0A2K0J0): KYGGECSKEHNTCT						
γAFP^A0A0A2K0J0#^	14	1556.69	6.74	+ 0.25	−1.486	2.87
*Aspergillus fischeri* (A1D8H8): EYKGECFTKDNTCK						
γAFP^A1D8H8^	14	1665.85	6.26	−1.5	−1.500	3.17
*Penicillium expansum* (A0A0A2K8K6): LYTGQCFKKDNICK						
γAFP^A0A0A2K8K6^	14	1660.97	8.82	+ 2.0	−0.607	1.71
*Penicillium rubens* (B6HWK0): KYTGKCTKSKNECK						
γAFP^B6HWK0^	14	1617.90	9.51	+ 4.0	−1.814	3.31
AFPg-group						
*Aspergillus giganteus* (P17737): TYNGKCYKKDNICK						
γAFP^P17737^	14	1677.95	9.18	+ 3.0	−1.450	2.75
*Aspergillus violaceofuscus* (A0A2V5H6U3): KYDGQCRKSENQCR						
γAFP^A0A2V5H6U3^	14	1714.89	8.86	+ 2.0	−2.271	5.30
*Aspergillus taichungensis* (A0A2J5HZT4): KYHGVCSKKNNSCK						
γAFP^A0A2J5HZT4^*^#^	14	1595.85	9.60	+ 4.25	−1.421	2.82
BP-group						
*Penicillium rubens* (B6HMF2): WKGGKCEVIGTRDCG (1), QSVGRCSAGGCD (2)						
γAFP1^B6HMF2^	15	1608.85	8.05	+ 1.0	−0.587	1.76
γAFP2^B6HMF2^	12	1139.22	5.82	0.00	−0.275	2.06
*Penicillium polonicum* (A0A1V6NXI2): WKGGKCQVIGSRDCG (1), EKGGHCPSNGCD (2)						
γAFP1^A0A1V6NXI2^	15	1593.84	8.90	+ 2.0	−0.593	1.73
γAFP2^A0A1V6NXI2^	12	1203.27	5.32	−0.75	−1.350	2.53
*Aspergillus candidus* (A0A2I2FBQ1): WKGGKCEVIGTRDCG (1), QNGGHCTGSGCD (2)						
γAFP1^A0A2I2FBQ1#^	15	1608.85	8.05	+ 1.0	−0.587	1.76
γAFP2^A0A2I2FBQ1#^	12	1135.15	5.08	−0.75	−0.983	2.1
*Aspergillus versicolor* (A0A1L9PRY7): WTGGQCEVVGTRDCG (1), DNGGHCTGSGCD (2)						
γAFP1^A0A1L9PRY7^	15	1567.71	4.37	−1.0	−0.367	1.62
γAFP2^A0A1L9PRY7^	12	1122.11	4.20	−1.75	−0.983	2.36
NFAP2-group						
*Aspergillus fischeri* (A1DBL3): VISGKCEWQGGQLNCI						
γAFP^A1DBL3#^	16	1735.01	5.96	0.00	0.075	0.43

After the species name, the UniProt database accession number is indicated. γAFPs selected for drug interaction analysis are marked with ^#^. γAFPs selected for comprehensive investigations are marked with asterisk. GRAVY: grand average of hydropathy value

### Antifungal Activity of γAFPs

**Fig. 2:**
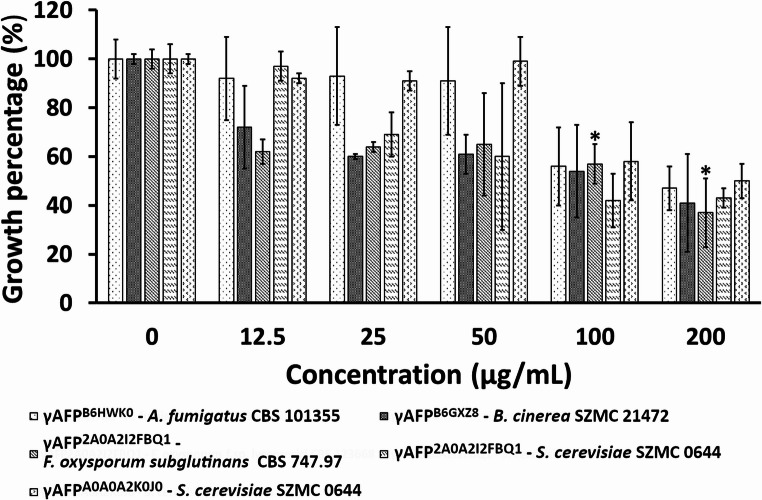
Growth percentages of fungi at various concentrations of the most effective γAFPs. Asterisk indicates not gradual dose-dependent inhibitory activity

The in vitro antifungal efficacy of γAFPs was evaluated against a diverse set of yeasts and molds, including human and plant pathogenic isolates, using a broth microdilution assay (Table 2). None of the tested γAFPs achieved complete growth inhibition at concentrations up to 200 µg/mL (minimum inhibitory concentration, MIC > 200 µg/mL). However, several γAFPs exhibited significant antifungal activity at the highest tested concentration (200 µg/mL). γAFP^B6HWK0^ displayed remarkable inhibitory effects against *A. fumigatus*. γAFP^B6GXZ8^, γAFP2^A0A2I2FBQ1^, and γAFP^A0A0A2K0J0^ demonstrated strong activity against *B. cinerea*, *F. subglutinans*, and S. *cerevisiae*, respectively (IP ≥ 50%, Table 2). Conversely, γAFP^A1D8H8^, γAFP1^B6HMF2^, γAFP2^A0A1V6NXI2^, and γAFP1^A0A1L9PRY7^ were considered inactive, exhibiting IPs between 0% and 14% ± 5% (Table 2). The remaining γAFPs inhibited the growth of at least one fungal species, with IPs of 25%–50% (Table 2). Statistical analysis of growth percentages at various concentrations of the most effective γAFPs revealed that their inhibitory activity was not concentration-dependent within the investigated concentration range (Fig. 2). Their efficacy remained constant beyond a certain threshold, and further increases in concentration did not yield statistically significant differences in growth reduction (*p* ≥ 0.05, Fig. 2). The effective concentrations were 100 µg/mL for γAFP^B6HWK0^ against *A. fumigatus* and γAFP^A0A0A2K0J0^ and γAFP2^A0A2I2FBQ1^ against *S. cerevisiae* and 25 µg/mL for γAFPB^6GXZ8^ against *B. cinerea* (Fig. 2). An exception was γAFP2^A0A2I2FBQ1^, which exhibited nonlinear concentration-dependent inhibition against *F. subglutinans* (Fig. 2).

**Table 2:** Growth inhibition percentages (IP) of 200 µg/mL antifungal-active γAFPs on the tested fungal isolates, relative to the untreated control cultures

*Fungus/Peptide*	*Aspergillus fumigatus* CBS 101355^+^	*Botrytis cinerea* SZMC 21472^++^	*Candida albicans* SC5314^+^	*Cladosporium herbarum* FSU 1148^++^	*Fusarium subglutinans* CBS 747.97^++^	Saccharomyces cerevisiae SZMC 0644
PAF-group IPs						
γAFP^A0A401KDC0^	**26 ± 12**	n.d.	10 ± 3	n.d.	n.d.	n.d.
γAFP^K9FGI7^	n.d.	n.d.	n.d.	**35 ± 2**	n.d.	n.d.
γAFP^B6GXZ8^*^#^	n.d.	**59 ± 20**	9 ± 12	14 ± 14	n.d.	**43 ± 1**
γAFP^A0A0A2K0J0#^	**37 ± 17**	**26 ± 3**	n.d.	n.d.	n.d.	**50 ± 7**
γAFP^A0A0A2K8K6^	12 ± 5	20 ± 8	14 ± 12	n.d.	n.d.	**33 ± 3**
γAFP^B6HWK0^	**53 ± 9**	18 ± 9	n.d.	n.d.	n.d.	18 ± 4
AFPg-group IPs						
γAFPP^17737^	9 ± 7	n.d.	n.d.	23 ± 18	n.d.	n.d.
γAFP^A0A2V5H6U3^	24 ± 0	20 ± 1	13 ± 0	18 ± 6	n.d.	15 ± 10
γAFP^A0A2J5HZT4^*^#^	22 ± 1	24 ± 14	13 ± 4	n.d.	**42 ± 8**	17 ± 3
BP-group IPs						
γAFP2^B6HMF2^	8 ± 3	**45 ± 3**	n.d.	n.d.	n.d.	20 ± 3
γAFP1^A0A1V6NXI2^	n.d.	**27 ± 1**	n.d.	**33 ± 1**	20 ± 4	7 ± 10
γAFP2^A0A2I2FBQ1#^	14 ± 2	n.d.	21 ± 7	n.d.	**63 ± 14**	**57 ± 4**
γAFP2^A0A1L9PRY7^	n.d.	11 ± 5	15 ± 4	n.d.	21 ± 6	**34 ± 4**
NFAP2-group IPs						
γAFP^A1DBL3#^	n.d.	n.d.	n.d.	n.d.	10 ± 4	**44 ± 4**

γAFPs selected for drug interaction analysis are marked with #. γAFPs selected for comprehensive investigations are marked with asterisk. ^+^: human pathogenic fungus, ^++^plant pathogenic fungus. n.d.: Growth inhibition was not detected. The growth inhibition percentage (IP) of the untreated control culture was defined as 0%

### Interactions between γAFPs and Conventional Drugs against *C. albicans* and *A. fumigatus*

The MICs of conventional antifungal drugs, namely FLC, AMB, MFG, and TRB, were determined using a broth microdilution assay against the human pathogenic fungi *C. albicans* and *A. fumigatus*. Under the applied test conditions, AMB (MIC = 1 µg/mL), MFG (MIC = 0.0156 µg/mL), and TRB (MIC = 1 µg/mL) effectively inhibited *C. albicans*, whereas FLC did not have such effects (MIC > 32 µg/mL). Complete growth inhibition of *A. fumigatus* was not achieved with AMB, MFG, TRB, or FLC (MIC > 32 µg/mL). Considering these MICs, AMB, MFG, and TRB were included in combination experiments against *C. albicans* to assess whether the presence of γAFPs could enhance their efficacy and lower the effective concentration, thereby reducing potential side effects associated with long-term, high-dose therapies [39]. Previous studies have demonstrated that co-administration of antimicrobial peptides can make resistant fungal strains susceptible to conventional antifungal drugs [40]. Given that *A. fumigatus* exhibits intrinsic resistance to FLC [41], FLC was tested in combination with γAFPs. In this experiment, conventional antifungal drugs were combined with an effective representative of each group of fungal AFPs with various physicochemical properties, i.e., γAFP^B6GXZ8^, γAFP^A0A0A2K0J0^, γAFP^A0A2J5HZT4^, γAFP2^A0A2I2FBQ1^, and γAFP^A1DBL3^ (Table 1). Most antifungal drug + γAFP combinations exhibited indifferent interactions, and no antagonistic effects were observed (data not shown). However, two notable exceptions emerged, as γAFP^B6GXZ8^ + TRB and γAFP^A0A2J5HZT4^ + FLC displayed synergy against *C. albicans* (Fig. S2) and *A. fumigatus* (Fig. S3), respectively. Subsequently, these synergistic combinations were included in further experiments, in which the highest IR and IP were detected below the individual MIC of the treatments (Figs. S2 and S3), namely 200 µg/mL γAFP^B6GXZ8^ + 0.5 µg/mL TRB against *C. albicans* and 200 µg/mL γAFP^A0A2J5HZT4^ + 32 µg/mL FLC against *A. fumigatus* (Table 3).

**Table 3:** Synergy between antifungal peptides (γAFP^B6GXZ8^, γAFP^A0A2J5HZT4^) and conventional antifungal drugs where the highest interaction ratios (IRs) and growth inhibitory percentages were detected below the individual MICs against *Candida albicans* or *Aspergillus fumigatus*. IRs were calculated according to the Abbott-formula

Combination	X and Y	I_e_	I_o_	IR	Type
*Candida albicans* SC5314					
γAFP^B6GXZ8^ (200 µg/mL) + TRB (0.5 µg/mL)	4 ± 3%	27 ± 7%	68 ± 7%	2.7 ± 0.7	Synergy
	24 ± 6%				
*Aspergillus fumigatus* CBS 101355					
γAFP^A0A2J5HZT4^ (200 µg/mL) + FLC (32 µg/mL)	24 ± 2%	24 ± 2%	70 ± 6%	3.0 ± 0.2	Synergy
	0 ± 0%				

FLC: fluconazole, I*e*: expected percentage inhibition, I_*0*_: observed percentage inhibition. IR: interaction ratio, TRB: terbinafine, X and Y: percentage inhibitions for the individual compounds used alone

### ECD Spectroscopy

**Fig. 3:**
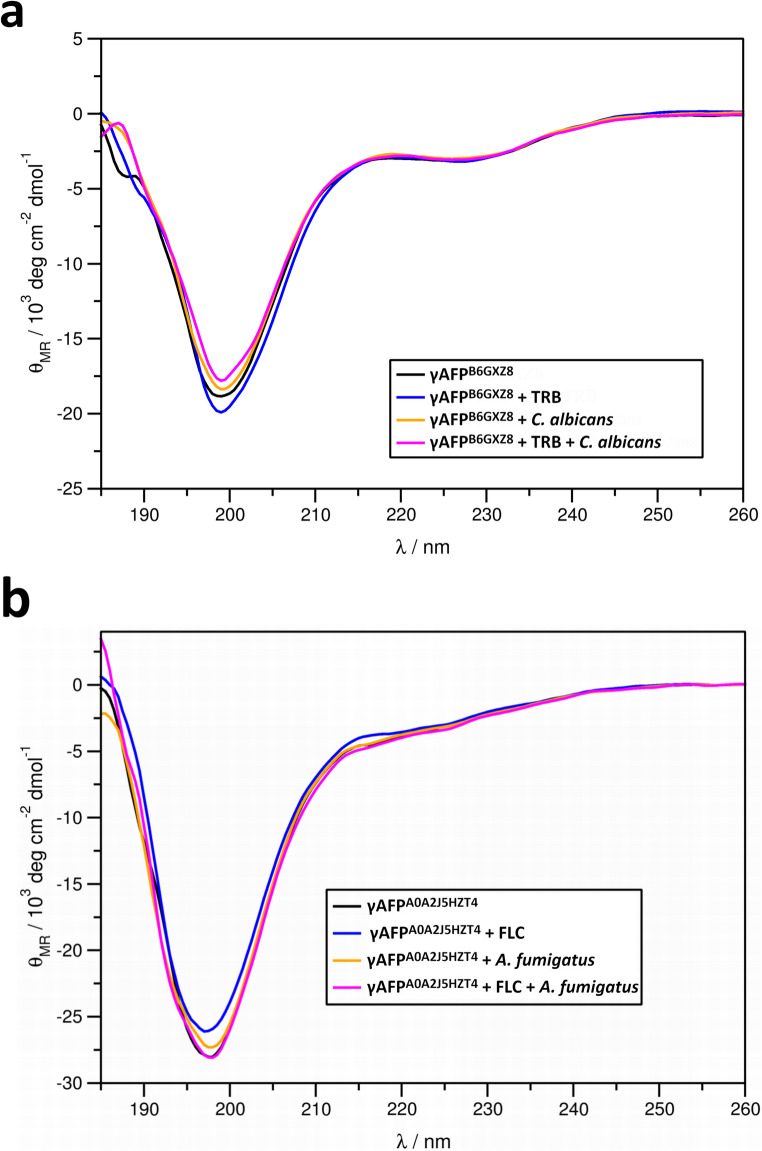
ECD spectra of γAFP^B6GXZ8^ (**a**) and γAFP^A0A2J5HZT4^ (**b**) in ddH_2_O, and in the presence of terbinafine (TRB), *Candida albicans* SC5314, and TRB and *C. albicans* SC3514 (**a**); and in the presence of fluconazole (FLC), *Aspergillus fumigatus* CBS 101355, and FLC and *A. fumigatus* CBS 101355 (**b**), respectively

Previously, we observed that synthetic γAFPs do not have an ordered structure, and a conformational change is not necessary for them to exert an antifungal effect [16, 19]. Both γAFP^B6GXZ8^ and γAFP^A0A2J5HZT4^ exhibited class D ECD spectra in all applied conditions, indicative of unordered structures, or, more precisely, high conformational flexibility and an ensemble of dynamic, fast interconverting structural states (Fig. 3). Spectral deconvolution of the ECD spectra indicated contributions from all canonical secondary structural elements; however, approximately 60% of the contributions emerged from the turn structures and noncanonical unordered conformations (Table S2). No considerable differences in spectral features and contributions were observed between the two peptides regardless of the applied experimental conditions. This indicates that the interactions of these peptides with fungal cells do not induce notable conformational reorganization. The obtained results support the in silico-predicted unordered structure of γAFP^A0A2J5HZT4^, but they contradict the predicted β-pleated conformation of γAFP^B6GXZ8^ (Fig. 1c).

### Fungal Cell-killing Efficacy of γAFP + Antifungal Drug Combinations

One of the primary antifungal mechanisms of antimicrobial peptides is plasma membrane disruption in the target fungus [42]. This membrane-compromising effect can be evaluated using PI staining. PI is a membrane-impermeant red fluorescent dye that selectively binds to nuclear and chromosomal DNA and only enters cells with compromised membrane integrity. FACS was utilized to quantify the cell-killing and membrane-disrupting capabilities of two potent γAFPs in combination with conventional antifungal agents. These combinations were compared with the standalone application of each compound to elucidate the observed synergistic interactions (Table 3). γAFP^B6GXZ8^ + TRB displayed significantly higher cell-killing efficacy than either compound alone (*p* = 6.5 × 10^⁻16^ for γAFP^B6GXZ8^, *p* = 2.4 × 10^⁻11^ for TRB) (Table 4). γAFP^A0A2J5HZT4^ + FLC also exhibited significantly enhanced cell-killing activity compared with FLC alone (*p* = 2.52 × 10^⁻12^). However, its efficacy was significantly lower than that of γAFP^A0A2J5HZT4^ alone (*p* = 3.52 × 10^⁻5^) (Table 4). Table S3 provides a detailed summary of the statistical analysis results.

**Table 4:** FACS analysis of cell death (propidium iodide positive cells/conidia, PI+) treated with γAFP, antifungal drug (TRB, FLC) and their combination

Treatment	PI+ (%)	*p*-value	
*Candida albicans* SC5314			
Untreated	0.2% ± 0.1	-	
γAFP^B6GXZ8^	46.3% ± 24.6	*p* = 6.5 × 10⁻^16^^*^	*p* = 0.072 ^ns^
TRB	44.5% ± 17.4	*p* = 2.4 × 10⁻^11^^*^	
γAFP^B6GXZ8^ + TRB	51.1% ± 23.9	-	
*Aspergillus fumigatus* CBS 101355			
Untreated	0.3% ± 0.18	-	
γAFP^A0A2J5HZT4^	15.1% ± 6.1	*p* = 3.52 × 10⁻^5^^*^	*p* = 2.48 × 10⁻^36^^+^
FLC	4.6% ± 1.3	*p* = 2.52 × 10⁻^12^^*^	
γAFP^A0A2J5HZT4^ + FLC	8.3% ± 0.9	-	

FLC: fluconazole, TRB: terbinafine

^*^: significant differences (*p* ≤ 0.05) between the standalone and combination treatment, ^+^: significant differences (*p* ≤ 0.05) between the standalone treatments, ^ns^: no significant difference

### SEM Analysis

**Fig. 4:**
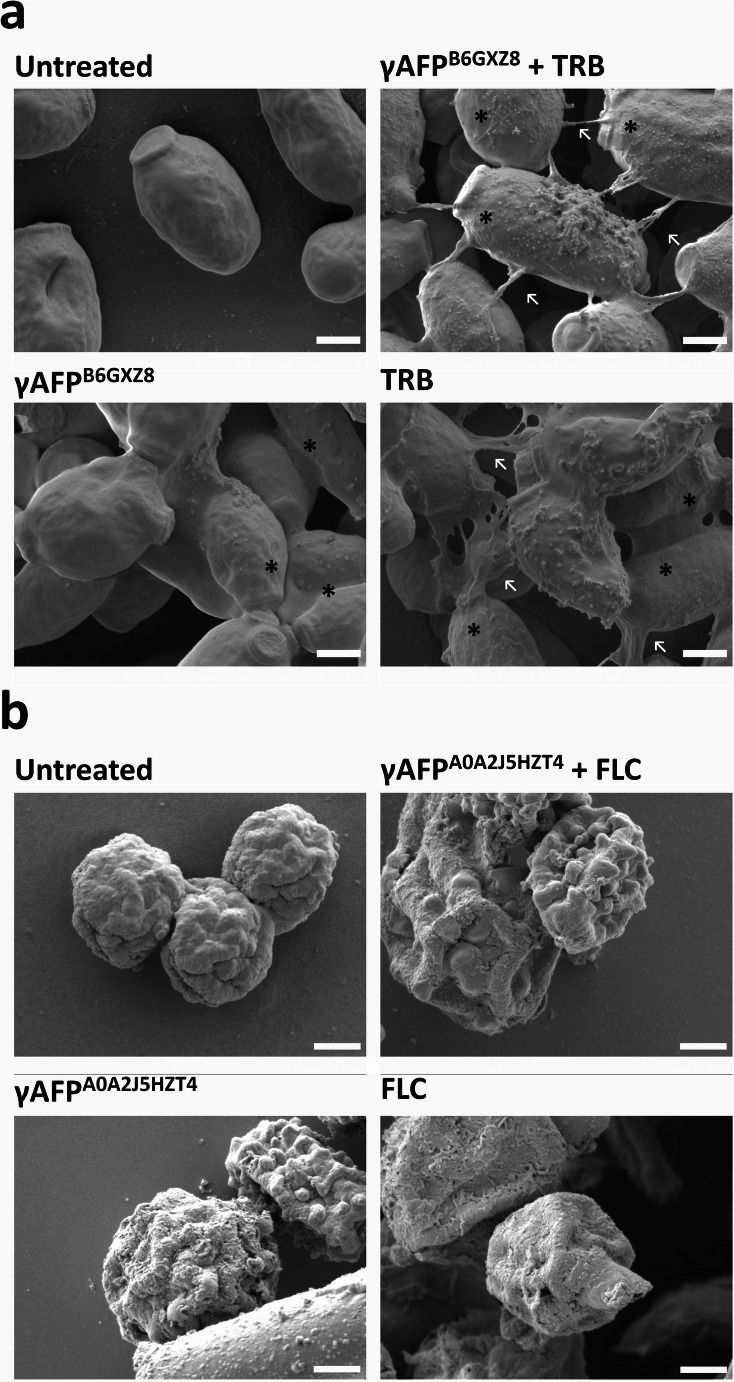
Scanning electron microscopy of *Candida albicans* SC5314 and *Aspergillus fumigatus* CBS 101355 treated with γAFP (γAFP^B6GXZ8^, γAFP^A0A2J5HZT4^), terbinafine (TRB) or fluconazole (FLC), and their combination (AFP^B6GXZ8^ + TRB and γAFP^A0A2J5HZT4^ + FLC) (4 × 10^6^ cells or conidia) in LCM, incubated at 30 °C for 16 h with shaking at 160 rpm for *C. albicans*, and under static conditions for *A. fumigatus*). Asterisks indicate representative *C. albicans* cells with membrane perturbation, while arrows the intercellular filamentous connections. Scale bars represent 1 μm

SEM further evidenced the antifungal activity of γAFP^B6GXZ8^, TRB, and their synergistic combination while revealing the associated morphological alterations (Fig. 4a). Treatment with γAFP^B6GXZ8^ induced notable surface changes in *C. albicans* cells, which were characterized by a coarse, dense surface with textured projections, deviating from the smooth, ovoid morphology typical of untreated yeast-phase cells. Some cells appeared partially deformed or aggregated, suggesting stress-induced responses, membrane disruption, or direct interaction with the peptide. Exposure to TRB induced similarly distinct alterations, including roughened and corrugated cell surfaces, indicative of membrane remodeling commonly observed under antifungal stress. In addition, some cells exhibited shrinkage, which could be the result of TRB-induced membrane permeabilization. Prominent cell aggregation and visible interfacial adhesion further indicated the initiation of a biofilm-like architecture under TRB pressure. Cells subjected to combination treatment showed morphological hallmarks attributable to both γAFP^B6GXZ8^ and TRB, including irregular surfaces with multiple protrusions and the presence of intercellular filamentous connections, suggestive of biofilm-associated growth.

SEM analysis revealed distinct morphological alterations in *A. fumigatus* conidia after antifungal treatments (Fig. 4b). Untreated spores maintained their characteristic smooth and rounded appearance, consistent with healthy, dormant conidia. Exposure to γAFP^A0A2J5HZT4^ alone resulted in moderate surface damage, suggesting membrane perturbation and partial structural compromise. The FLC-treated conidia showed minimal deformation, reflecting the limited efficacy of azoles against dormant fungal forms. Notably, the combination of γAFP^A0A2J5HZT4^ and FLC produced extensive morphological disruption, including collapsed and irregular conidial structures.

### Hemolytic Activity and Toxicity of γAFP + Antifungal Drug Combinations

The therapeutic application of antimicrobial peptides is often limited by their potential to induce hemolysis in red blood cells [43]. The well-established *G. mellonella* acute toxicity assay serves as a reliable model for assessing the in vivo harmful effects of drug candidates [44]. To evaluate the hemolytic activity and toxic effects of the combinations, γAFP^B6GXZ8^ + TRB and γAFP^A0A2J5HZT4^ + FLC were tested in vitro on sheep blood agar plates and in vivo using *G. mellonella* larvae. None of the γAFPs and antifungal drugs, either alone or in combination, caused hemolysis or significantly reduced larval survival (Fig. S4). In contrast, treatment with Triton X-100 resulted in both effects (Fig. S4). These findings indicate that γAFPs can be safely used alone and in combination with antifungal drugs for therapeutic purposes.

### In Vivo Therapeutic Potential of γAFP + antifungal Drug Combinations

**Fig. 5:**
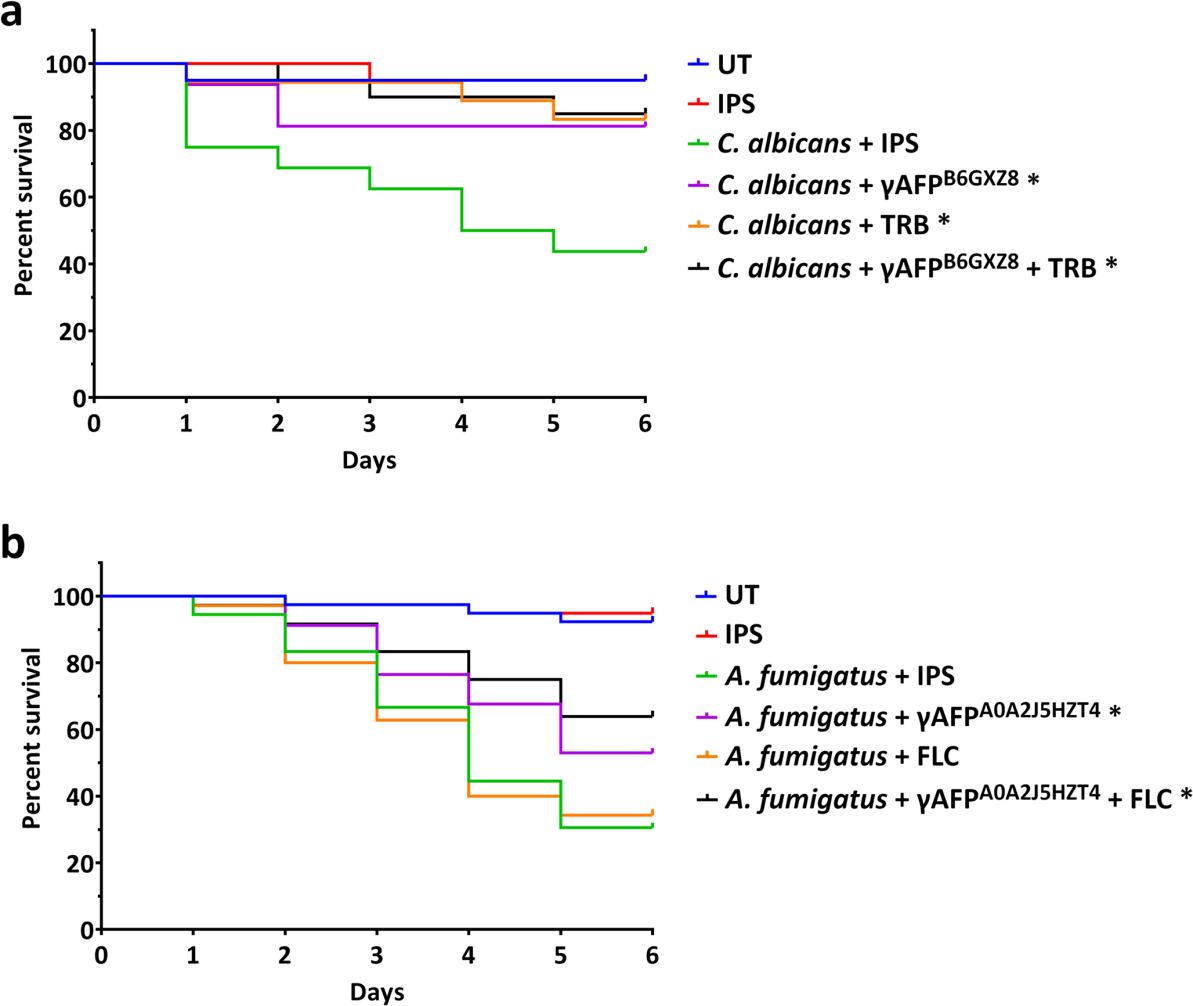
In vivo therapeutic potential of γAFP^B6GXZ8^, γAFP^A0A2J5HZT4^, terbinafine (TRB), fluconazole (FLC), γAFP^B6GXZ8^ + TRB and γAFP^A0A2J5HZT4^ + FLC combinations in *Galleria mellonella* larval infection model. Larvae were infected with *Candida albicans* SC5314 then treated with, γAFP^B6GXZ8^, TRB, or their synergistic combination (**a**). Larvae were infected with *Aspergillus fumigatus* CBS 101355 then treated with γAFP^A0A2J5HZT4^, FLC or their synergistic combination (**b**). UT: not infected and untreated control, IPS: insect physiological saline-treated control, *C. albicans* + IPS: *C. albicans* infected and IPS-treated (untreated), *C. albicans* + γAFP^B6GXZ8^: *C. albicans* infected and γAFP^B6GXZ8^-treated (200 µg/mL), *C. albicans* + TRB: *C. albicans* infected and TRB-treated (1 µg/mL), *C. albicans* + γAFP^B6GXZ8^ + TRB: *C. albicans* infected and γAFP^B6GXZ8^ (200 µg/mL) - TRB (0.5 µg/mL) combination-treated and, *A. fumigatus* + IPS: *A. fumigatus* infected and IPS-treated (untreated), *A. fumigatus* + γAFP^A0A2J5HZT4^: *A. fumigatus* infected and γAFP^A0A2J5HZT4^-treated (200 µg/mL), *A. fumigatus* + FLC: *A. fumigatus* infected and FLC-treated (32 µg/mL), *A. fumigatus* + γAFP^A0A2J5HZT4^ + FLC: *A. fumigatus* infected and + γAFP^A0A2J5HZT4^ (200 µg/mL) – FLC (32 µg/mL) combination-treated groups. IPS: insect physiological saline-treated control. *: *p* ≤ 0.05 from both Log rank (Mantel-Cox) and Gehan-Breslow-Wilcoxon tests in compared to the infected *(C. albicans* or *A. fumigatus* + IPS), not treated group

The in vivo therapeutic potential of γAFP^B6GXZ8^, TRB, and their synergistic combination was evaluated in *G. mellonella* larvae infected with *C. albicans* SC5314 (Fig. 5a). Infection significantly reduced larval survival compared with the findings in the IPS-treated control group (*p* = 0.0001). Similar findings were obtained when infected larvae were treated with either γAFP^B6GXZ8^ or TRB alone (*p* = 0.0047 and *p* = 0.0336, respectively). However, a significant reduction in larval survival was not observed when γAFP^B6GXZ8^ and TRB were applied in combination (*p* = 0.0533). Compared with the findings in infected and untreated larvae, the combination treatment exerted a statistically more pronounced therapeutic effect (*p* = 0.024 for γAFP^B6GXZ8^ and *p* = 0.0015 for TRB vs. *p* = 0.0004). Survival was prolonged and increased in the order of γAFP^B6GXZ8^ (*p* = 0.0245) < TRB (*p* = 0.001) < γAFP^B6GXZ8^ + TRB combination (*p* = 0.0002); however, no statistically significant differences were detected among these groups (*p* > 0.05) (Table S4). These findings suggest that the combined application of TRB and γAFPB^6GXZ8^ provides a superior therapeutic effect than either treatment alone and potentially prevents the development of infection.

The in vivo therapeutic efficacy of γAFP^A0A2J5HZT4^ + FLC against *A. fumigatus* CBS 101355 was evaluated (Fig. 5b). Compared with the results in the IPS-treated control group, a significant decrease in larval survival was observed upon infection with *A. fumigatus* conidia (*p* < 0.0001). γAFP^A0A2J5HZT4^ and FLC, either alone (*p* < 0.0001) or in combination (*p* = 0.0009), did not prevent this decline. In comparison to the findings in the infected but untreated group, treatment with FLC alone did not significantly affect larval survival (*p* = 0.9503). However, γAFP^A0A2J5HZT4^ alone and in combination with FLC prolonged (*p* = 0.0375 and *p* = 0.0045, respectively) and increased (*p* = 0.0442 and *p* = 0.0063, respectively) survival, with the combination yielding a statistically stronger outcome. Based on the observed p-values, no significant difference was found between the effect of γAFP^A0A2J5HZT4^ and in combination with FLC (*p* > 0.05). However, compared with the effects of FLC monotherapy, the combined treatment significantly improved survival (*p* = 0.0085, respectively) (Table S5). These findings indicate that γAFP^A0A2J5HZT4^ possesses moderate therapeutic potential as a standalone treatment that can be further enhanced in the presence of FLC and the ability to prolong larval survival.

Figure S1 presents a flow chart that summarizes the main results.

## Discussion

Most synthetic peptides encompassing the native γ-core region of fungal AFPs have exhibited no antifungal activity [16, 45, 46], suggesting that only highly hydrophilic and positively charged γ-core peptides possess antifungal activity [13, 46, 47]. Our study refined these observations by systematically examining 19 γAFPs, and found that antifungal activity among γAFPs is primarily governed by the interplay between net positive charge and hydrophilicity, rather than by either factor alone. Peptides that showed a moderately positive net charge and hydrophilic character displayed the highest inhibitory potential (Table 1). In contrast, highly charged or hydrophobic variants showed diminished activity (Table 1), underscoring the importance of achieving an optimal charge-hydropathy balance. This pattern is consistent with previous findings in *P. chrysogenum* PAF and *P. expansum* PeAfpB, where amino acid substitutions influencing the net charge enhanced antifungal efficacy up to a threshold beyond which peptide aggregation or nonspecific interactions reduced activity [13, 14, 16, 49]. Our study expands on these models by identifying a quantitative relationship between the net charge-to-hydropathy ratio and the antifungal efficacy, as shown in Tables 1 and 2. Peptides with ratios that fell within a defined range exhibited inhibition against yeast and filamentous fungal isolates, suggesting that this physicochemical parameter may serve as a predictive metric for rational γAFP design.

Among the peptides screened, γAFP^B6GXZ8^ (from *P. rubens*) and γAFP^A0A2J5HZT4^ (from *A. taichungensis*) emerged as the most potent antifungal candidates (Table 1). Both peptides exhibited pronounced in vitro activity (IP ≥ 40%) (Table 2) and induced measurable membrane damage, as inferred from PI uptake (Table 4), FACS analysis, and surface alterations observed under SEM (Fig. 4). However, these data provide only indirect evidence of membrane perturbation. They do not directly confirm membrane permeabilization at the biophysical level, and we therefore acknowledge that the proposed membranolytic mechanism remains inferential. Specifically, no liposome leakage assays, SYTOX Green uptake, electrophysiological analyses, or ion-conductance measurements were performed in this study. Future experiments employing calcein- or ANTS/DPX-loaded liposomes, stopped-flow fluorimetry, or molecular dynamics simulations will be required to directly validate γAFP-induced pore formation or lipid packing disruption. Despite these limitations, the observed PI uptake patterns are consistent with the membranolytic mechanism proposed for several γ-core derivatives in earlier studies [16, 49, 50].

A major novel finding of this work is the demonstration of peptide–drug synergy between γAFPs and conventional antifungal agents. Checkerboard microdilution assays revealed that γAFP^B6GXZ8^ exhibited a strong synergistic interaction with TRB against *C. albicans*, while γAFP^A0A2J5HZT4^ acted synergistically FLC against *A. fumigatus* (Table 3, Figs. S2 and S3). Both TRB and FLC target enzymes essential for ergosterol biosynthesis, the squalene epoxidase and lanosterol 14-α-demethylase, respectively [51]. Since γAFPs disrupt fungal plasma membranes (Fig. 4), their combination with drugs that inhibit membrane component synthesis likely amplifies membrane destabilization, leading to enhanced fungicidal effects. This mechanism is supported by the significantly increased proportion of PI-positive cells observed upon combined γAFP^B6GXZ8^ + TRB treatment (Table 4 and Table S3), indicating increased disruption of the plasma membrane. Conversely, the γAFP^A0A2J5HZT4^ + FLC combination showed no significant in vitro or in vivo enhancement under our experimental conditions (Table 4; Fig. 5b), likely due to differences in fungal lipid composition and the intrinsic azole tolerance of *A. fumigatus* [41]. ECD spectroscopy revealed that none of the γAFPs underwent significant structural rearrangements upon interaction with fungal cells or antifungal agents, confirming that these short peptides remain largely disordered in solution. This intrinsic flexibility may facilitate dynamic adaptation to diverse membrane environments, as also proposed for other linear γ-core-derived peptides [16, 49].

The in vivo* G. mellonella* infection model further validated the antifungal efficacy and indicated low toxicity of the two lead γAFPs. Peptide administration did not cause significant larval mortality in the absence of infection, confirming biocompatibility (Fig. S4). In infected larvae, γAFP^B6GXZ8^ effectively prevented the progression of *C. albicans* infection, and statistical analyses indicated that its activity was further enhanced in the presence of terbinafine (TRB) (Fig. 5a and Table S4). In contrast, γAFP^A0A2J5HZT4^ alone or in combination with fluconazole (FLC) produced only moderate survival prolongation in larvae infected with *A. fumigatus* without complete protection (Fig. 5b). Although the observed improvements in survival rates in the presence of γAFP + antifungal drug combinations were modest, statistical analyses indicated higher therapeutic efficacy (Tables S4 and S5). These results suggest that γAFP + antifungal drug combinations may enhance therapeutic efficacy under certain conditions, particularly when conventional antifungal agents show limited standalone activity (e.g., FLC against *A. fumigatus*). However, we acknowledge that all in vivo data presented in this study rely exclusively on the *G. mellonella* model, which (while suitable for initial screening) does not replicate mammalian immunity, pharmacokinetics, or toxicity. Therefore, any implications regarding therapeutic applicability must be interpreted with caution, and confirmation in appropriate mammalian infection models will be essential to establish true translational relevance. By presenting these data with appropriate caution, we emphasize both the promising potential and the current limitations of γAFP-based combination therapies.

Beyond in vivo efficacy, several translational constraints must also be considered. First, γAFPs are short linear peptides and may be susceptible to host and fungal proteases. Although their high positive charge and partial disorder can confer some proteolytic tolerance, systematic stability profiling in serum, fungal secretomes, and host-like protease mixtures will be required to estimate in vivo half-lives. Second, production costs for synthetic peptides remain higher than for small-molecule antifungals; however, the short length (≤ 15 aa) of γAFPs and their straightforward synthesis suggest that cost barriers may be moderate compared with larger therapeutic peptides. Third, resistance development is generally less frequent for membrane-active peptides, but cannot be excluded. Monitoring for adaptive membrane remodeling, efflux modulation, or peptide sequestration will therefore be essential in future studies.

Importantly, even though γAFPs exhibited only partial inhibition in vitro (Table 2), such activity remains biologically relevant. Partial growth suppression can substantially reduce the viable fungal cell or spore population, thereby lowering the infectious burden and facilitating host immune clearance. Moreover, this sublethal inhibition can potentiate the efficacy of conventional antifungal agents when applied in combination therapy, as evidenced by the synergistic effects observed in this study. From a therapeutic standpoint, such combinations may also help mitigate resistance development by decreasing the pool of persistent fungal cells that often survive monotherapy and drive relapse. In addition, employing γAFPs as adjuvants could enable dose reduction of conventional antifungals, minimizing the risk of adverse effects associated with prolonged or high-dose treatments.

Collectively, our findings underscore the translational potential of γAFPs as complementary agents in antifungal regimens aimed at enhancing efficacy while improving safety and resistance management. The net charge-hydropathy relationship identified here offers a rational framework for designing optimized γAFPs with enhanced potency and selectivity. Moreover, the demonstrated synergy between γAFPs and conventional antifungals (particularly the γAFP^B6GXZ8^ + TRB combination) suggests that such peptide + drug formulations may hold promise for both topical and systemic applications. While TRB is primarily used for superficial *Candida* infections and fluconazole is not a first-line agent for aspergillosis [41, 52], these experimental pairings highlight the potential therapeutic scope of γAFP-based adjuvant strategies.

## Conclusions

Numerous studies have demonstrated that synthetic peptides encompassing the evolutionarily conserved γ-core motifs of antifungal proteins can exhibit antifungal activity and may hold therapeutic relevance, particularly as modulators of existing treatments. Based on the findings of this study, we propose that the antifungal efficacy of γAFPs is influenced primarily by the magnitude of the net positive charge and hydrophilicity, with the charge-to-hydropathy ratio emerging as a potential predictor of activity rather than a fully validated determinant. Importantly, the quadratic regression analysis underlying the proposed charge-to-hydropathy relationship (Fig. S5) was performed on a limited peptide set (*n* = 19) and therefore should be considered exploratory. The lack of external validation constrains the generalizability of this model, and larger, independent γAFP libraries will be required to confirm or refine its predictive utility. Nevertheless, the preliminary trend (showing that net charge critically shapes the hydropathy profile of γAFPs and yields a minimum GRAVY value at approximately + 4.2) suggests that an optimal balance between electrostatic interactions and solubility may facilitate membrane interaction and disruption. Among the 19 peptides derived from Eurotiomycetes γ-core regions, only a subset conformed to this physicochemical window and exhibited moderate antifungal activity (Tables 1 and 2), indicating that γ-core motifs likely support structural stability and folding rather than directly encoding antifungal potency. When active, these peptides most likely exert their effects through plasma membrane disruption. Together, our findings indicate that γAFPs are best positioned not as independent antifungal agents, but as well-tolerated synergistic peptide adjuvants capable of enhancing the efficacy of existing antifungal drugs and providing structurally tractable scaffolds for rational optimization. However, several translational factors require careful consideration before clinical development can be pursued. γAFPs are short linear peptides and may be susceptible to degradation by host and fungal proteases, and their stability under physiologically relevant conditions remains untested. Although synthetic peptide production costs exceed those of small-molecule antifungals, the short length of γAFPs suggests that manufacturing demands may be manageable. While membrane-active peptides typically show reduced propensity for resistance development, adaptive responses, such as altered lipid composition or peptide sequestration, cannot be excluded. Finally, because all in vivo findings in this study derive from *G. mellonella*, validation in mammalian infection models will be essential to fully assess efficacy, toxicity, and pharmacokinetics. Future studies should address these limitations through physicochemical model–guided structure–activity optimization, systematic stability and protease-resistance profiling, cost-feasibility analysis, and comprehensive evaluation in mammalian fungal infection models to establish translational relevance.

## Supplementary Information

Below is the link to the electronic supplementary material.

Supplementary Material 1

## Data Availability

The authors declare that all data supporting the findings of this study are available within the article and its supplementary information files and are available from the corresponding author upon request.
