# Supplementary material for: Screening the γ-core Motif Peptides of Ascomycetous Antifungal Proteins for Antifungal Activity and Potential Therapeutic Applicability
Source: Probiotics Antimicrob Proteins. 2026 Jan 6;18(5):6693–710. doi: 10.1007/s12602-025-10890-y (PMC13369176; doi:10.1007/s12602-025-10890-y)
Supplement: Supplementary file 1 — Supplementary Material 1 [file 12602_2025_10890_MOESM1_ESM.docx]

**Supplementary Information**

**Screening the γ-core motifs of ascomycetous antifungal proteins for antifungal activity and potential therapeutic applicability**

John K Karemera^1,2^, Györgyi Váradi^3^, Gábor Bende^1,2,4^, Richárd Merber^1,2^, Kinga Dán^1,2^, Csaba Papp^1^, Attila Farkas^5^, Gergely Maróti^5^, Gábor K. Tóth^3^, Attila Borics^6^, László Galgóczy^1^

^1^ Department of Biotechnology and Microbiology, Faculty of Science and Informatics, University of Szeged, Szeged, Hungary

^2^ Doctoral School of Biology, Faculty of Science and Informatics, University of Szeged, Szeged, Hungary

^3^ Department of Medical Chemistry, Albert Szent-Györgyi Medical School, University of Szeged, Szeged, Hungary

^4^ Department of Theoretical Health Sciences and Health Management, Faculty of Health Sciences and Social Studies, University of Szeged, Szeged, Hungary

^5^ Institute of Plant Biology, HUN-REN Biological Research Centre, Szeged, Hungary

^6^ Institute of Biochemistry, HUN-REN Biological Research Centre, Szeged, Hungary

Correspondence: László Galgóczy (galgoczi@bio.u-szeged.hu)

John K Karemera (ORCID): 0009-0008-4316-6904

Györgyi Váradi (ORCID): 0000-0001-7907-8908

Gábor Bende (ORCID): 0000-0003-4571-9775

Kinga Dán (ORCID): 0009-0003-1900-6517

Csaba Papp (ORCID): 0000-0003-4450-0667

Attila Farkas (ORCID): 0000-0002-0413-8248

Gergely Maróti (ORCID): 0000-0002-3705-0461

Attila Borics (ORCID): 0000-0002-6331-3536

László Galgóczy (ORCID): 0000-0002-6976-8910

**Table S1.** Amino acid sequences of antifungal proteins (AFPs) from Eurotiomycetes involved in the study.

| Peptide | Length  (aa) | Mw  (Da) | Theoretical  pI | Net charge  (pH = 7.0) | GRAVY | Boman index  (kcal/mol) |
| --- | --- | --- | --- | --- | --- | --- |
| PAF-group | | | | | | |
| *Aspergillus awamori* (A0A401KDC0)  MQLTSIAIILFAAMGAIATPITAEADNLVAREAELS**KYGGECSVEHNTCT**YLKGGKDHIVSCPSAANLRCKTERHHCEYDEHHKTVDCQTPV | | | | | | |
| *Penicillium digitatum* (K9FGI7)  MQITSIAIILFTAMGAVANPIATASDDLDARDVQLS**KYGGQCSLKHNTCT**YLKGGRNVIVNCGSAANKRCKSDRHHCEYDEHHRRVDCQTPV | | | | | | |
| *Penicillium rubens* (B6GXZ8)  MHITSIAIVFFAAMGAVASPIATESDDLDARDVQLS**KFGGECSLKHNTCT**YLKGGKNHVVNCGSAANKKCKSDRHHCEYDEHHKRVDCQTPV | | | | | | |
| *Penicillium expansum* (A0A0A2K0J0)  MQITRIAIFLFAAMGAVASPIVAESRDVDAQALS**KYGGECSKEHNTCT**YRKDGKDHIVKCPSADNKKCKTDRHHCEYDDHHKTVDCQTPV | | | | | | |
| *Neosartorya fischeri* (A1D8H8)  MQITKISLFLFVGIGVVASPIHAESDGLNARAVNAADL**EYKGECFTKDNTCK**YKIDGKTYLAKCPSAANTKCEKDGNKCTYDSYNRKVKCDFRH | | | | | | |
| *Penicillium expansum* (A0A0A2K8K6)  MQITKIALFLFAAMGAVASPIEAEAESGINARAENGANV**LYTGQCFKKDNICK**YKVNGKQNIAKCPSAANKRCEKDKNKCTFDSYDRKVTCDFRK | | | | | | |
| *Penicillium rubens* (B6HWK0)  MQITTVALFLFAAMGGVATPIESVSNDLDARAEAGVLA**KYTGKCTKSKNECK**YKNDAGKDTFIKCPKFDNKKCTKDNNKCTVDTYNNAVDCD | | | | | | |
| AFPg-group | | | | | | |
| *Aspergillus giganteus* (P17737)  MKFVSLASLGFALVAALGAVATPVEADSLTAGGLDARDESAVLA**TYNGKCYKKDNICK**YKAQSGKTAICKCYVKKCPRDGAKCEFDSYKGKCYC | | | | | | |
| *Aspergillus violaceofuscus* (A0A2V5H6U3)  MKISPVSIGFILLAAMGVAATPLNHAESVGVRSENNVQV**KYDGQCRKSENQCR**YTAQSGRTAICKCQFRKCSKDGAKCNFDSYNRDCNCY | | | | | | |
| *Aspergillus taichungensis* (A0A2J5HZT4)  MQLISLASMGLVLFAAVGAVASPVDNNALDIDNNLEVRDEAASLI**KYHGVCSKKNNSCK**FKGQNGKTSFCHCKFKKCGKENNKCHFDSYNRDCKCI | | | | | | |
| BP-group | | | | | | |
| *Penicillium rubens* (B6HMF2)  MKVTALLFTLMAATAVSASVLDTRDTCGGGYGVDQRRTNSPCQASNGDRHFC**GCDRTGIVECKGGKW**TEIQ**DCGGASCRGVSQ**GGARC | | | | | | |
| *Penicillium polonicum* (A0A1V6NXI2)  MKFTAMLFTLMAATAVSASVLETRDGCGSAYGPDQRRTNSPCQSSNGNKQYC**GCDRSGIVQCKGGKW**TEVQD**CGNSPCHGGKE**GGALC | | | | | | |
| *Aspergillus candidus* (A0A2I2FBQ1)  MKLIAIVCTLMAAASVSASTIEARDTCGAGYGGDQRRTNSPCASSNGDRHFC**GCDRTGIVECKGGKW**TEVK**DCGSGTCHGGNQ**GAAQC | | | | | | |
| *Aspergillus versicolor* (A0A1L9PRY7)  MKLSIFFATLLAAAVSAGSVLEARDTCGAGYGGDQRRTNSACDASNGDRHFC**GCDRTGVVECQGGTW**TEIS**DCGSGTCHGGND**GGAQC | | | | | | |
| NFAP2-group | | | | | | |
| *Neosartorya fischeri* (A1DBL3)  MHLSTALFSAIALLAATQVIGASVEVPRDVAAIQIATSPYYACNCPNNCKHKKGSGCKYHSGPSDKSK**VISGKCEWQGGQLNCI**AT | | | | | | |

After the species name, the UniProt database accession number is indicated. Amino acid sequence regions used for γAFP design are indicated with bold and underlined letters in the primary AFP structure.

**Table S2.** Secondary structural contributions to the observed ECD spectra of γAFP^B6GXZ8^ and γAFP^A0A2J5HZT4^ obtained from spectral deconvolution.

|  | Helix1 | Helix2 | Strand1 | Strand2 | Turns | Unordered | Total |
| --- | --- | --- | --- | --- | --- | --- | --- |
| γAFP^B6GXZ8^ | 0.01 | 0.08 | 0.18 | 0.11 | 0.26 | 0.36 | 1 |
| γAFP^B6GXZ8^ + TRB | 0.02 | 0.09 | 0.18 | 0.11 | 0.26 | 0.35 | 1.01 |
| γAFP^B6GXZ8^ + *C. albicans* | 0.01 | 0.08 | 0.19 | 0.11 | 0.25 | 0.36 | 1 |
| γAFP^B6GXZ8^ + TRB + *C. albicans* | 0.01 | 0.08 | 0.19 | 0.11 | 0.25 | 0.35 | 0.99 |
| γAFP^A0A2J5HZT4^ | -.01 | 0.10 | 0.17 | 0.11 | 0.25 | 0.37 | 0.99 |
| γAFP^A0A2J5HZT4^ + FLC | -.01 | 0.08 | 0.19 | 0.11 | 0.24 | 0.37 | 0.98 |
| γAFP^A0A2J5HZT4^ + *A. fumigatus* | -.01 | 0.10 | 0.16 | 0.10 | 0.25 | 0.39 | 0.99 |
| γAFP^A0A2J5HZT4^ + FLC + *A. fumigatus* | -.01 | 0.09 | 0.18 | 0.10 | 0.24 | 0.39 | 0.99 |

**Table S3.** Statistical analysis (Pearson’s chi-squared test and the Phi coefficient) of FACS results.

| Comparison | p-value | Significance  (p ≤ 0.05) | φ (phi) | Effect size |
| --- | --- | --- | --- | --- |
| *Candida albicans* SC5341 | | | | |
| Combination vs. γAFPB^6GXZ8^ | 2.4 × 10^–11^ | Yes | 0.0421 | Small |
| Combination vs. TRB | 6.5 × 10^–16^ | Yes | 0.0521 | Small |
| γAFPB^6GXZ8^ vs. TRB | 0.072 | No | 0.018 | Negligible |
| *Aspergillus fumigatus* CBS 101355 | | | | |
| Combination vs. γAFP^A0A2J5HZT4^ | 3.5 × 10^–5^ | Yes | 0.0414 | Small |
| Combination vs. FLC | 2.5 × 10^–12^ | Yes | 0.0695 | Small |
| γAFP^A0A2J5HZT4^ vs. FLC | 2.5 × 10^–36^ | Yes | 0.125 | Medium |

FLC: fluconazole, TRB: terbinafine

**Table S4.** Statistical analysis of *Candida albicans* SC5314-infected larval survival treated with γAFP^B6GXZ8^, terbinafine (TRB), and their combination.

| IPS *vs.* *C. albicans* SC5314 + IPS | | |
| --- | --- | --- |
| Log-rank (Mantel-Cox) test | | |
| Chi square | 24.2 |  |
| df | 1 |  |
| P value | <0.0001 |  |
| P value summary | ******** |  |
| Are the survival curves sig different? | Yes |  |
| Gehan-Breslow-Wilcoxon test | | |
| Chi square | 24.12 |  |
| df | 1 |  |
| P value | <0.0001 |  |
| P value summary | ******** |  |
| Are the survival curves sig different? | Yes |  |
| Median survival | | |
| IPS | Undefined |  |
| *C. albicans* + IPS | 4.5 |  |
| Hazard Ratio (Mantel-Haenszel) | B/C | C/B |
| Ratio (and its reciprocal) | 0.09657 | 10.36 |
| 95% CI of ratio | 0.03805 to 0.2451 | 4.080 to 26.28 |
| Hazard Ratio (logrank) | B/C | C/B |
| Ratio (and its reciprocal) | 0.06881 | 14.53 |
| 95% CI of ratio | 0.02793 to 0.1696 | 5.898 to 35.81 |
| IPS *vs.* *C. albicans* SC5314 + γAFP^B6GXZ8^ | | |
| Log-rank (Mantel-Cox) test | | |
| Chi square | 7.999 |  |
| df | 1 |  |
| P value | 0.0047 |  |
| P value summary | ****** |  |
| Are the survival curves sig different? | Yes |  |
| Gehan-Breslow-Wilcoxon test | | |
| Chi square | 8.148 |  |
| df | 1 |  |
| P value | 0.0043 |  |
| P value summary | ****** |  |
| Are the survival curves sig different? | Yes |  |
| Median survival | | |
| IPS | Undefined |  |
| *C. albicans* + γAFP^B6GXZ8^ | Undefined |  |
| Hazard Ratio (Mantel-Haenszel) | B/D | D/B |
| Ratio (and its reciprocal) | 0.1887 | 5.3 |
| 95% CI of ratio | 0.05939 to 0.5993 | 1.669 to 16.84 |
| Hazard Ratio (logrank) | B/D | D/B |
| Ratio (and its reciprocal) | 0.1531 | 6.53 |
| 95% CI of ratio | 0.04889 to 0.4796 | 2.085 to 20.45 |
| IPS *vs.* *C. albicans* SC5314 + TRB | | |
| Log-rank (Mantel-Cox) test | | |
| Chi square | 4.516 |  |
| df | 1 |  |
| P value | 0.0336 |  |
| P value summary | ***** |  |
| Are the survival curves sig different? | Yes |  |
| Gehan-Breslow-Wilcoxon test | | |
| Chi square | 4.521 |  |
| df | 1 |  |
| P value | 0.0335 |  |
| P value summary | ***** |  |
| Are the survival curves sig different? | Yes |  |
| Median survival | | |
| IPS | Undefined |  |
| *C. albicans* + TRB | Undefined |  |
| Hazard Ratio (Mantel-Haenszel) | B/E | E/B |
| Ratio (and its reciprocal) | 0.256 | 3.907 |
| 95% CI of ratio | 0.07284 to 0.8995 | 1.112 to 13.73 |
| Hazard Ratio (logrank) | B/E | E/B |
| Ratio (and its reciprocal) | 0.2204 | 4.536 |
| 95% CI of ratio | 0.06366 to 0.7633 | 1.310 to 15.71 |
| IPS *vs.* *C. albicans* SC5314 + γAFP^B6GXZ8^ + TRB | | |
| Log-rank (Mantel-Cox) test | | |
| Chi square | 3.736 |  |
| df | 1 |  |
| P value | 0.0533 |  |
| P value summary | ns |  |
| Are the survival curves sig different? | No |  |
| Gehan-Breslow-Wilcoxon test | | |
| Chi square | 3.71 |  |
| df | 1 |  |
| P value | 0.0541 |  |
| P value summary | ns |  |
| Are the survival curves sig different? | No |  |
| Median survival | | |
| IPS | Undefined |  |
| *C. albicans* + γAFP^B6GXZ8^ + TRB | Undefined |  |
| Hazard Ratio (Mantel-Haenszel) | B/F | F/B |
| Ratio (and its reciprocal) | 0.2906 | 3.442 |
| 95% CI of ratio | 0.08297 to 1.017 | 0.9828 to 12.05 |
| Hazard Ratio (logrank) | B/F | F/B |
| Ratio (and its reciprocal) | 0.2477 | 4.037 |
| 95% CI of ratio | 0.07172 to 0.8557 | 1.169 to 13.94 |
| *C. albicans* SC5314 +IPS *vs.* *C. albicans* SC5314 +γAFP^B6GXZ8^ | | |
| Log-rank (Mantel-Cox) test | | |
| Chi square | 5.093 |  |
| df | 1 |  |
| P value | 0.024 |  |
| P value summary | ***** |  |
| Are the survival curves sig different? | Yes |  |
| Gehan-Breslow-Wilcoxon test | | |
| Chi square | 5.057 |  |
| df | 1 |  |
| P value | 0.0245 |  |
| P value summary | ***** |  |
| Are the survival curves sig different? | Yes |  |
| Median survival | | |
| *C. albicans* + IPS | 4.5 |  |
| *C. albicans* + γAFP^B6GXZ8^ | Undefined |  |
| Hazard Ratio (Mantel-Haenszel) | C/D | D/C |
| Ratio (and its reciprocal) | 2.472 | 0.4045 |
| 95% CI of ratio | 1.126 to 5.426 | 0.1843 to 0.8878 |
| Hazard Ratio (logrank) | C/D | D/C |
| Ratio (and its reciprocal) | 2.273 | 0.4399 |
| 95% CI of ratio | 1.078 to 4.793 | 0.2086 to 0.9274 |
| *C. albicans* SC5314 + IPS *vs.* *C. albicans* SC5314 + TRB | | |
| Log-rank (Mantel-Cox) test | | |
| Chi square | 10.13 |  |
| df | 1 |  |
| P value | 0.0015 |  |
| P value summary | ****** |  |
| Are the survival curves sig different? | Yes |  |
| Gehan-Breslow-Wilcoxon test | | |
| Chi square | 10.75 |  |
| df | 1 |  |
| P value | 0.001 |  |
| P value summary | ****** |  |
| Are the survival curves sig different? | Yes |  |
| Median survival | | |
| *C. albicans* + IPS | 4.5 |  |
| *C. albicans* + TRB | Undefined |  |
| Hazard Ratio (Mantel-Haenszel) | C/E | E/C |
| Ratio (and its reciprocal) | 3.791 | 0.2638 |
| 95% CI of ratio | 1.668 to 8.614 | 0.1161 to 0.5994 |
| Hazard Ratio (logrank) | C/E | E/C |
| Ratio (and its reciprocal) | 3.375 | 0.2963 |
| 95% CI of ratio | 1.540 to 7.396 | 0.1352 to 0.6494 |
| *C. albicans* SC5314 +IPS *vs.* *C. albicans* SC5314 + γAFP^B6GXZ8^ + TRB | | |
| Log-rank (Mantel-Cox) test | | |
| Chi square | 12.7 |  |
| df | 1 |  |
| P value | 0.0004 |  |
| P value summary | ******* |  |
| Are the survival curves sig different? | Yes |  |
| Gehan-Breslow-Wilcoxon test | | |
| Chi square | 13.75 |  |
| df | 1 |  |
| P value | 0.0002 |  |
| P value summary | ******* |  |
| Are the survival curves sig different? | Yes |  |
| Median survival | | |
| *C. albicans* + IPS | 4.5 |  |
| *C. albicans*+γAFP^B6GXZ8^ + TRB | Undefined |  |
| Hazard Ratio (Mantel-Haenszel) | C/F | F/C |
| Ratio (and its reciprocal) | 4.515 | 0.2215 |
| 95% CI of ratio | 1.970 to 10.35 | 0.09665 to 0.5075 |
| Hazard Ratio (logrank) | C/F | F/C |
| Ratio (and its reciprocal) | 3.857 | 0.2592 |
| 95% CI of ratio | 1.739 to 8.558 | 0.1168 to 0.5751 |
| *C. albicans* SC5314 + γAFP^B6GXZ8^ *vs.* *C. albicans* SC5314 + TRB | | |
| Log-rank (Mantel-Cox) test | | |
| Chi square | 0.7003 |  |
| df | 1 |  |
| P value | 0.4027 |  |
| P value summary | ns |  |
| Are the survival curves sig different? | No |  |
| Gehan-Breslow-Wilcoxon test | | |
| Chi square | 0.8203 |  |
| df | 1 |  |
| P value | 0.3651 |  |
| P value summary | ns |  |
| Are the survival curves sig different? | No |  |
| Median survival | | |
| *C. albicans* + γAFPB^6GXZ8^ | Undefined |  |
| *C. albicans* + TRB | Undefined |  |
| Hazard Ratio (Mantel-Haenszel) | D/E | E/D |
| Ratio (and its reciprocal) | 1.5 | 0.6666 |
| 95% CI of ratio | 0.5803 to 3.878 | 0.2579 to 1.723 |
| Hazard Ratio (logrank) | D/E | E/D |
| Ratio (and its reciprocal) | 1.47 | 0.6804 |
| 95% CI of ratio | 0.5816 to 3.714 | 0.2693 to 1.719 |
| *C. albicans* SC5314 + γAFP^B6GXZ8^ *vs.* *C. albicans* SC5314 + γAFPB^6GXZ8^ + TRB | | |
| Log-rank (Mantel-Cox) test | | |
| Chi square | 1.252 |  |
| df | 1 |  |
| P value | 0.2632 |  |
| P value summary | ns |  |
| Are the survival curves sig different? | No |  |
| Gehan-Breslow-Wilcoxon test | | |
| Chi square | 1.493 |  |
| df | 1 |  |
| P value | 0.2217 |  |
| P value summary | ns |  |
| Are the survival curves sig different? | No |  |
| Median survival | | |
| *C. albicans* + γAFP^B6GXZ8^ | Undefined |  |
| *C. albicans* + γAFP^B6GXZ8^ + TRB | Undefined |  |
| Hazard Ratio (Mantel-Haenszel) | D/F | F/D |
| Ratio (and its reciprocal) | 1.724 | 0.58 |
| 95% CI of ratio | 0.6640 to 4.476 | 0.2234 to 1.506 |
| Hazard Ratio (logrank) | D/F | F/D |
| Ratio (and its reciprocal) | 1.672 | 0.5982 |
| 95% CI of ratio | 0.6571 to 4.252 | 0.2352 to 1.522 |
| *C. albicans* SC5314 + TRB *vs.* *C. albicans* SC5314 + γAFP^B6GXZ8^ + TRB | | |
| Log-rank (Mantel-Cox) test | | |
| Chi square | 0.07 |  |
| df | 1 |  |
| P value | 0.7913 |  |
| P value summary | ns |  |
| Are the survival curves sig different? | No |  |
| Gehan-Breslow-Wilcoxon test | | |
| Chi square | 0.08884 |  |
| df | 1 |  |
| P value | 0.7657 |  |
| P value summary | ns |  |
| Are the survival curves sig different? | No |  |
| Median survival | | |
| *C. albicans* + TRB | Undefined |  |
| *C. albicans* + γFPB6GXZ8 + TRB | Undefined |  |
| Hazard Ratio (Mantel-Haenszel) | E/F | F/E |
| Ratio (and its reciprocal) | 1.145 | 0.8735 |
| 95% CI of ratio | 0.4203 to 3.118 | 0.3207 to 2.379 |
| Hazard Ratio (logrank) | E/F | F/E |
| Ratio (and its reciprocal) | 1.138 | 0.8785 |
| 95% CI of ratio | 0.4263 to 3.039 | 0.3290 to 2.346 |

**Table S5.** Statistical analysis of *Aspergillus fumigatus* CBS 101355-infected larval survival treated with γAFP^A0A2J5HZT4^, fluconazole (FLC), and their combination.

| IPS *vs.* *A. fumigatus* CBS 101355 + IPS | | |
| --- | --- | --- |
| Log-rank (Mantel-Cox) test | | |
| Chi square | 33.76 |  |
| df | 1 |  |
| P value | <0,0001 |  |
| P value summary | ******** |  |
| Are the survival curves sig different? | Yes |  |
| Gehan-Breslow-Wilcoxon test | | |
| Chi square | 31.61 |  |
| df | 1 |  |
| P value | <0,0001 |  |
| P value summary | ******** |  |
| Are the survival curves sig different? | Yes |  |
| Median survival | | |
| IPS | Undefined |  |
| *A. fumigatus* + IPS | 4 |  |
| Hazard Ratio (Mantel-Haenszel) | B/C | C/B |
| Ratio (and its reciprocal) | 0.09077 | 11.02 |
| 95% CI of ratio | 0,0404 to 0,2039 | 4,904 to 24,75 |
| Hazard Ratio (logrank) | B/C | C/B |
| Ratio (and its reciprocal) | 0.0544 | 18.38 |
| 95% CI of ratio | 0,02523 to 0,1173 | 8,524 to 39,64 |
| IPS *vs.* *A. fumigatus* CBS 101355 + γAFP^A0A2J5HZT4^ | | |
| Log-rank (Mantel-Cox) test | | |
| Chi square | 16.12 |  |
| df | 1 |  |
| P value | <0,0001 |  |
| P value summary | ******** |  |
| Are the survival curves sig different? | Yes |  |
| Gehan-Breslow-Wilcoxon test | | |
| Chi square | 15.56 |  |
| df | 1 |  |
| P value | <0,0001 |  |
| P value summary | ******** |  |
| Are the survival curves sig different? | Yes |  |
| Median survival | | |
| IPS | Undefined |  |
| *A. fumigatus* + γAFP^A0A2J5HZT4^ | Undefined |  |
| Hazard Ratio (Mantel-Haenszel) | B/D | D/B |
| Ratio (and its reciprocal) | 0.1398 | 7.153 |
| 95% CI of ratio | 0,05351 to 0,3653 | 2,738 to 18,69 |
| Hazard Ratio (logrank) | B/D | D/B |
| Ratio (and its reciprocal) | 0.09573 | 10.45 |
| 95% CI of ratio | 0,03769 to 0,2431 | 4,113 to 26,53 |
| IPS *vs.* *A. fumigatus* CBS 101355 + FLC | | |
| Log-rank (Mantel-Cox) test | | |
| Chi square | 30.71 |  |
| df | 1 |  |
| P value | <0,0001 |  |
| P value summary | ******** |  |
| Are the survival curves sig different? | Yes |  |
| Gehan-Breslow-Wilcoxon test | | |
| Chi square | 29.09 |  |
| df | 1 |  |
| P value | <0,0001 |  |
| P value summary | ******** |  |
| Are the survival curves sig different? | Yes |  |
| Median survival | | |
| IPS | Undefined |  |
| *A. fumigatus* + FLC | 4 |  |
| Hazard Ratio (Mantel-Haenszel) | B/E | E/B |
| Ratio (and its reciprocal) | 0.09203 | 10.87 |
| 95% CI of ratio | 0,03958 to 0,214 | 4,673 to 25,27 |
| Hazard Ratio (logrank) | B/E | E/B |
| Ratio (and its reciprocal) | 0.05922 | 16.89 |
| 95% CI of ratio | 0,02665 to 0,1316 | 7,599 to 37,53 |
| IPS *vs.* *A. fumigatus* CBS 101355 + γAFP^A0A2J5HZT4^+ FLC | | |
| Log-rank (Mantel-Cox) test | | |
| Chi square | 10.95 |  |
| df | 1 |  |
| P value | 0.0009 |  |
| P value summary | ******* |  |
| Are the survival curves sig different? | Yes |  |
| Gehan-Breslow-Wilcoxon test | | |
| Chi square | 10.65 |  |
| df | 1 |  |
| P value | 0.0011 |  |
| P value summary | ****** |  |
| Are the survival curves sig different? | Yes |  |
| Median survival | | |
| IPS | Undefined |  |
| *A. fumigatus* + FLC + γAFP^A0A2J5HZT4^ | Undefined |  |
| Hazard Ratio (Mantel-Haenszel) | B/F | F/B |
| Ratio (and its reciprocal) | 0.1737 | 5.757 |
| 95% CI of ratio | 0,06158 to 0,4899 | 2,041 to 16,24 |
| Hazard Ratio (logrank) | B/F | F/B |
| Ratio (and its reciprocal) | 0.1257 | 7.954 |
| 95% CI of ratio | 0,04546 to 0,3477 | 2,876 to 22 |
| *A. fumigatus* CBS 101355 + IPS *vs.* *A. fumigatus* CBS 101355 + γAFP^A0A2J5HZT4^ | | |
| Log-rank (Mantel-Cox) test | | |
| Chi square | 4.328 |  |
| df | 1 |  |
| P value | 0.0375 |  |
| P value summary | ***** |  |
| Are the survival curves sig different? | Yes |  |
| Gehan-Breslow-Wilcoxon test | | |
| Chi square | 4.047 |  |
| df | 1 |  |
| P value | 0.0442 |  |
| P value summary | ***** |  |
| Are the survival curves sig different? | Yes |  |
| Median survival | | |
| *A. fumigatus* + IPS | 4 |  |
| *A. fumigatus* + γAFP^A0A2J5HZT4^ | Undefined |  |
| Hazard Ratio (Mantel-Haenszel) | C/D | D/C |
| Ratio (and its reciprocal) | 2.048 | 0.4883 |
| 95% CI of ratio | 1,042 to 4,023 | 0,2486 to 0,9594 |
| Hazard Ratio (logrank) | C/D | D/C |
| Ratio (and its reciprocal) | 1.816 | 0.5507 |
| 95% CI of ratio | 0,9828 to 3,355 | 0,298 to 1,018 |
| *A. fumigatus* CBS 101355 + IPS *vs.* *A. fumigatus* CBS 101355 + FLC | | |
| Log-rank (Mantel-Cox) test | | |
| Chi square | 0.003893 |  |
| df | 1 |  |
| P value | 0.9503 |  |
| P value summary | ns |  |
| Are the survival curves sig different? | No |  |
| Gehan-Breslow-Wilcoxon test | | |
| Chi square | 0.02008 |  |
| df | 1 |  |
| P value | 0.8873 |  |
| P value summary | ns |  |
| Are the survival curves sig different? | No |  |
| Median survival | | |
| *A. fumigatus* + IPS | 4 |  |
| *A. fumigatus* + FLC | 4 |  |
| Ratio (and its reciprocal) | 1 | 1 |
| 95% CI of ratio | 0,5676 to 1,762 | 0,5676 to 1,762 |
| Hazard Ratio (Mantel-Haenszel) | C/E | E/C |
| Ratio (and its reciprocal) | 1.021 | 0.9798 |
| 95% CI of ratio | 0,5377 to 1,937 | 0,5162 to 1,86 |
| Hazard Ratio (logrank) | C/E | E/C |
| Ratio (and its reciprocal) | 1.016 | 0.9842 |
| 95% CI of ratio | 0,5768 to 1,79 | 0,5588 to 1,734 |
| *A. fumigatus* CBS 101355 + IPS *vs.* *A. fumigatus* CBS 101355 + γAFP^A0A2J5HZT4^ +FLC | | |
| Log-rank (Mantel-Cox) test | | |
| Chi square | 8.061 |  |
| df | 1 |  |
| P value | 0.0045 |  |
| P value summary | ****** |  |
| Are the survival curves sig different? | Yes |  |
| Gehan-Breslow-Wilcoxon test | | |
| Chi square | 7.467 |  |
| df | 1 |  |
| P value | 0.0063 |  |
| P value summary | ****** |  |
| Are the survival curves sig different? | Yes |  |
| Median survival | | |
| *A. fumigatus* + IPS | 4 |  |
| *A. fumigatus* + FLC + γAFP^A0A2J5HZT4^ | Undefined |  |
| Hazard Ratio (Mantel-Haenszel) | C/F | F/C |
| Ratio (and its reciprocal) | 2.732 | 0.366 |
| 95% CI of ratio | 1,365 to 5,468 | 0,1829 to 0,7325 |
| Hazard Ratio (logrank) | C/F | F/C |
| Ratio (and its reciprocal) | 2.381 | 0.4201 |
| 95% CI of ratio | 1,256 to 4,513 | 0,2216 to 0,7963 |
| *A. fumigatus* CBS 101355 + γAFP^A0A2J5HZT4^ *vs.* *A. fumigatus* CBS 101355 + FLC | | |
| Log-rank (Mantel-Cox) test | | |
| Chi square | 3.651 |  |
| df | 1 |  |
| P value | 0.056 |  |
| P value summary | ns |  |
| Are the survival curves sig different? | No |  |
| Gehan-Breslow-Wilcoxon test | | |
| Chi square | 4.012 |  |
| df | 1 |  |
| P value | 0.0452 |  |
| P value summary | *** |  |
| Are the survival curves sig different? | Yes |  |
| Median survival | | |
| *A. fumigatus* + γAFP^A0A2J5HZT4^ | Undefined |  |
| *A. fumigatus* + FLC | 4 |  |
| Hazard Ratio (Mantel-Haenszel) | D/E | E/D |
| Ratio (and its reciprocal) | 0.5094 | 1.963 |
| 95% CI of ratio | 0,2551 to 1,017 | 0,9828 to 3,92 |
| Hazard Ratio (logrank) | D/E | E/D |
| Ratio (and its reciprocal) | 0.5713 | 1.75 |
| 95% CI of ratio | 0,304 to 1,073 | 0,9316 to 3,289 |
| *A. fumigatus* CBS 101355 + γAFP^A0A2J5HZT4^ *vs.* *A. fumigatus* CBS 101355 + FLC + γAFP^A0A2J5HZT4^ | | |
| Log-rank (Mantel-Cox) test | | |
| Chi square | 0.6143 |  |
| df | 1 |  |
| P value | 0.4332 |  |
| P value summary | ns |  |
| Are the survival curves sig different? | No |  |
| Gehan-Breslow-Wilcoxon test | | |
| Chi square | 0.5733 |  |
| df | 1 |  |
| P value | 0.449 |  |
| P value summary | ns |  |
| Are the survival curves sig different? | No |  |
| Median survival | | |
| *A. fumigatus* + γAFP^A0A2J5HZT4^ | Undefined |  |
| *A. fumigatus* + FLC + γAFP^A0A2J5HZT4^ | Undefined |  |
| Hazard Ratio (Mantel-Haenszel) | D/F | F/D |
| Ratio (and its reciprocal) | 1.361 | 0.7345 |
| 95% CI of ratio | 0,6294 to 2,945 | 0,3396 to 1,589 |
| Hazard Ratio (logrank) | D/F | F/D |
| Ratio (and its reciprocal) | 1.317 | 0.7593 |
| 95% CI of ratio | 0,6358 to 2,729 | 0,3665 to 1,573 |
| *A. fumigatus* CBS 101355 +FLC *vs.* *A. fumigatus* CBS 101355 + FLC + γAFP^A0A2J5HZT4^ | | |
| Log-rank (Mantel-Cox) test | | |
| Chi square | 6.928 |  |
| df | 1 |  |
| P value | 0.0085 |  |
| P value summary | ****** |  |
| Are the survival curves sig different? | Yes |  |
| Gehan-Breslow-Wilcoxon test | | |
| Chi square | 7.064 |  |
| df | 1 |  |
| P value | 0.0079 |  |
| P value summary | ****** |  |
| Are the survival curves sig different? | Yes |  |
| Median survival | | |
| *A. fumigatus* + FLC | 4 |  |
| *A. fumigatus* + FLC +γ AFP^A0A2J5HZT4^ | Undefined |  |
| Hazard Ratio (Mantel-Haenszel) | E/F | F/E |
| Ratio (and its reciprocal) | 2.604 | 0.384 |
| 95% CI of ratio | 1,277 to 5,311 | 0,1883 to 0,7832 |
| Hazard Ratio (logrank) | E/F | F/E |
| Ratio (and its reciprocal) | 2.272 | 0.4402 |
| 95% CI of ratio | 1,176 to 4,388 | 0,2279 to 0,8504 |


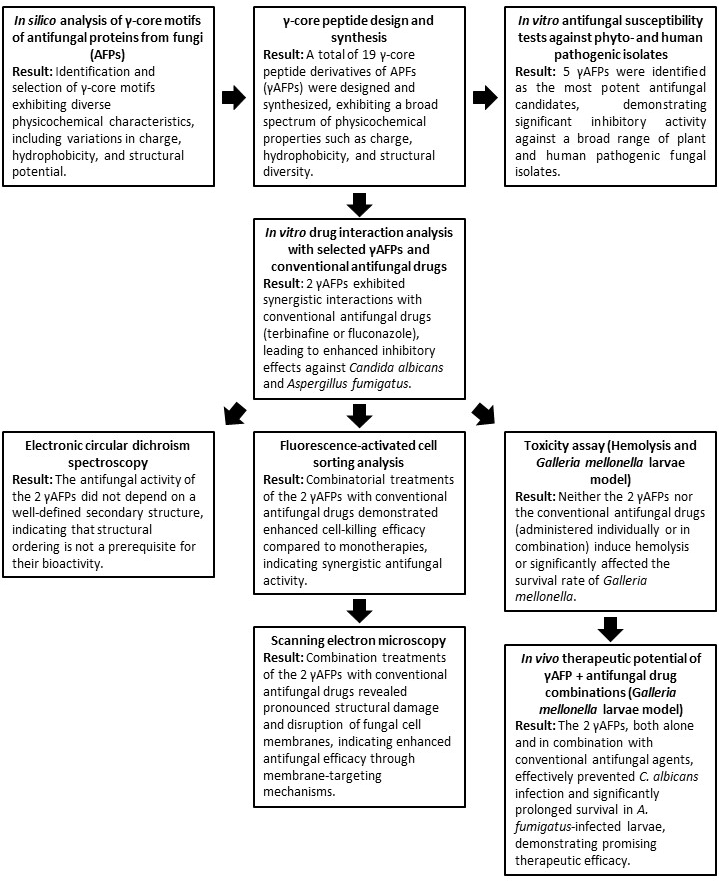


**Fig. S1.** Flowchart illustrating the study design and corresponding results.

| ***Candida albicans* SC3514** | | | | | | | | | | | | |
| --- | --- | --- | --- | --- | --- | --- | --- | --- | --- | --- | --- | --- |
| **γ^AFPB6GXZ8^ +**  **TRB** | **400 µg/ml** | **200 µg/ml** | **100 µg/ml** | **50 µg/ml** | **25 µg/ml** | **12.5 µg/ml** | **6.25 µg/ml** | **3.125 µg/ml** | **1.56 µg/ml** | **0.78 µg/ml** | **0.39 µg/ml** | **0** |
| **2 µg/ml** | 94±1.2%  IR:1.02±0.0 | 94±1%  IR:1.02±0.01 | 93±0.8%  IR: 1.02±0.01 | 94±1.3%  IR: 1.02±0.03 | 94±1.9%  IR: 1.0±0.04 | 93±1.9%  IR: 1.02±0.01 | 93±1.5%  IR: 1.02±0.01 | 93±0.6%  IR: 1.02±0.04 | 92±0.5%  IR: 1.0±0.01 | 92±1%  IR: 1.0±0.01 | 92±1%  IR: 1.01±0.01 | 92±1.3% |
| **1 µg/ml** | 94±0.6%  IR: 1.01±0.01 | 95±1%  IR: 1.03±0.01 | 93±0.5%  IR: 1.01±0.01 | 93±1.2%  IR: 1.01±0.015 | 92±0.5%  IR: 1.03±0.05 | 93±0.6%  IR: 1.01±0 | 93±1.3%  IR: 1.01±0.01 | 93±0.6%  IR: 1.01±0.01 | 92±0.6%  IR:1.0±X0.0 | 92±0.5%  IR: 0.99±0.01 | 92±0.5%  IR: 1.0±0 | 92±0.5% |
| **0.5 µg/ml** | 73±4.8%  IR: 2.83±1.08 | **68±7.1%**  **IR: 2.7±0.73** | 55±6.8%  IR: 2.08±0.45 | 52±6.7%  IR: 2.02±0.49 | 47±6.6%  IR: 1.68±0.35 | 40±2.4%  IR: 1.58±0.29 | 36±4.5%  IR: 1.48±0.33 | 33±5.9%  IR: 1.17±0.01 | 32±4.9%  IR: 1.06±0.24 | 28±7%  IR: 0.93±0.1 | 24±6.3%  IR: 0.94±0.27 | 24±6.1% |
| **0.25 µg/ml** | 33±9.1%  IR: 1.89±0.23 | 24±2.4%  IR:  1.83±1.0 | 20±2.2%  IR: 1.29±0.3 | 24±10.4%  IR: 1.61±0.5 | 16±8.5%  IR: 0.96±0.31 | 21±4.9%  IR: 1.56±0.37 | 18±3%  IR: 1.32±0.32 | 20±2.4%  IR: 1.19±0.28 | 13±2.8%  IR: 0.71±0.25 | 17±2.6%  IR: 0.88±0.15 | 18±2.2%  IR: 1.19±0.15 | 12±2.8% |
| **0.125 µg/ml** | 18±6.8%  IR: 0.92±0.34 | 14±5.1%  IR: 0.81±0.14 | 21±4%  IR: 1.16±0.25 | 18±4.2%  IR: 1.04±0.2 | 24±2.8%  IR: 1.36±0.31 | 11±2.6%  IR: 0.63±0.03 | 14±3.1%  IR: 0.84±0.14 | 17±2.8%  IR: 0.86±0.08 | 16±2.6%  IR: 0.75±0.23 | 11±3.3%  IR: 0.47±0.05 | 20±3.1%  IR: 1.21±0.24 | 15±2.6% |
| **0.0625 µg/ml** | 12±3.7%  IR: 0.8±0.23 | 10±3.3%  IR: 0.77±0.1 | 9±2.6%  IR: 0.62±0.07 | 17±2.6%  IR: 1.40±0.53 | 12±3.1%  IR: 0.76±0.1 | 14±2.9%  IR: 1.23±0.33 | 15±2.6%  IR: 1.28±0.39 | 16±2.8%  IR: 1.07±0.22 | 14±3.3%  IR: 0.89±0.45 | 6±2.9%  IR: 0.34±0.09 | 13±3.1%  IR: 0.93±0.22 | 9±2.3% |
| **0.03125 µg/ml** | 14±2.6%  IR: 0.99±0.32 | 15±2.8%  IR: 1.42±0.55 | 13±2.6%  IR: 0.97±0.22 | 13±2.6%  IR: 1.15±0.4 | 11±2.8%  IR: 0.93±0.32 | 15±2.9%  IR: 1.49±0.51 | 13±3.3%  IR: 1.19±0.36 | 12±2.6%  IR: 0.82±0.15 | 13±2.9%  IR: 0.87±0.39 | 11±2.4%  IR: 0.7±0.14 | 10±1.3%  IR: 0.7±0.21 | 9±2.8% |
| **0 µg/ml** | 6±3.7% | 4±2.9% | 5±2.4% | 4±2.9% | 5±3.7% | 3±2.5% | 3±3.9% | 7±2.9% | 9±6.4% | 9±3.4% | 4±2.8% | 0±0% |

**Fig S2.** Inhibition percentages (IP, %) of *Candida albicans* SC5314 in combinatorial application of γ^AFPB6GXZ8^ + terbinafine (TRB). Interaction ratio (IR) calculated according to the Abbot-formula presented below IP. Red cells indicate synergy (IR >1.5), while orange cells additive interaction. The untreated control was defined as 100% of growth.

| ***Aspergillus fumigatus* CBS 101355** | | | | | | | | | | | | |
| --- | --- | --- | --- | --- | --- | --- | --- | --- | --- | --- | --- | --- |
| **γ^AFPB6GXZ8^ +**  **FLC** | **400 µg/ml** | **200 µg/ml** | **100 µg/ml** | **50 µg/ml** | **25 µg/ml** | **12.5 µg/ml** | **6.25 µg/ml** | **3.125 µg/ml** | **1.56 µg/ml** | **0.78 µg/ml** | **0.39 µg/ml** | **0** |
| **64 µg/ml** | 58±8.2%  IR: 1.59±0.46 | 65±7.2%  IR: 2.51±0.23 | 54±2.6%  IR: 2.14±0.44 | 13±11.3%  IR: 0.37±0.29 | 23±4.3%  IR: 1.77±1.43 | 23±3.6%  IR: 0.88±0.4 | 8±10%  IR: 0.72±0.84 | 4±4.7%  IR: 0.4±0.59 | 1±1%  IR: 0.1±0.1 | 4±4.8%  IR: 0.21±0.24 | 7±2.5%  IR: 0.22±0.25 | 4±4.3% |
| **32 µg/ml** | 62±5.7%  IR: 1.91±0.67 | **70±5.9%**  **IR: 2.99±0.15** | 65±3.3%  IR: 2.84±0.28 | 33±25.8%  IR: 1.33±1.18 | 48±1%  IR: 4.03±3.36 | 39±14.8%  IR: 1.51±0.21 | 19±8.5%  IR: 2.55±3.14 | 00±0%  IR: 0.0±0 | 4±4.2%  IR: 0.1±0.17 | 00±0.0%  IR: 0.0±0 | 00±00%  IR: 0.0±0 | 00±0.0% |
| **16 µg/ml** | 67±3.2%  IR: 1.79±0.56 | 73±4.8%  IR: 2.79±0.42 | 71±0.8%  IR: 2.79±0.56 | 38±29.2%  IR: 1.35±1.13 | 43±8.5%  IR: 3.05±2.73 | 36±12.1%  IR: 1.59±1.09 | 22±10.7%  IR: 2.33±1.93 | 38±19.8%  IR: 3.35±3.12 | 29±8.1%  IR: 2.0±2.14 | 37±19.1%  IR: 2.52±3.51 | 14±15.9%  IR: 1.1±2.2 | 4±2.9% |
| **8 µg/ml** | 58±8.5%  IR: 1.67±0.81 | 72±5.2%  IR: 2.88±0.28 | 64±1.3%  IR: 2.74±0.49 | 33±37.8%  IR: 1.38±1.64 | 46±21.4%  IR: 4.01±3.88 | 49±17.9%  IR: 2.22±1.61 | 49±7.3%  IR: 7.13±6.11 | 18±10.9%  IR: 1.32±0.51 | 21±5.9%  IR: 2.15±2.69 | 31±16%  IR: 3.59±4.9 | 16±23.1%  IR: 3.1±6.12 | 2±2.6% |
| **4 µg/ml** | 59±4.1%  IR: 1.46±0.46 | 72±5%  IR: 2.85±0.36 | 66±4.6%  IR: 2.41±0.24 | 22±25.4%  IR: 0.67±0.82 | 54±0.5%  IR: 2.36±1.41 | 55±3.3%  IR: 1.95±0.84 | 32±11.5%  IR: 4.2±1.81 | 12±12.2%  IR: 0.51±0.34 | 32±17.3%  IR: 3.29±2.42 | 33±20.7%  IR: 3.5±2.95 | 16±10.3%  IR: 1.67±1.91 | 4±2.9% |
| **2 µg/ml** | 53±5.2%  IR: 1.33±0.47 | 76±3.9%  IR: 2.66±0.37 | 67±2.9%  IR: 2.48±0.53 | 62±1.2%  IR: 1.88±0.2 | 39±11.6%  IR: 1.86±1.6 | 44±3.7%  IR: 1.5±0.62 | 30±10%  IR: 5.33±3.51 | 12±13.3%  IR: 0.32±0.38 | 30±11.1%  IR: 3.9±4.1 | 4±5.1%  IR: 0.56±0.82 | 11±3.8%  IR: 1.22±0.79 | 6±11.6% |
| **1 µg/ml** | 63±1.3%  IR: 1.62±0.53 | 76±2.8%  IR: 3.26±0.23 | 72±3.2%  IR: 3.14±0.37 | 56±7.3%  IR: 2.1±0.69 | 53±4.8%  IR: 4.18±3.35 | 55±3.4%  IR: 2.33±0.95 | 27±25.4%  IR: 0.31±0.62 | 10±4.1%  IR: 0.78±0.97 | 10±8.5%  IR: 0.0±0 | 15±13%  IR: 0.15±0.21 | 20±12.7%  IR: 0.41±0.73 | 00±0.0% |
| **0 µg/ml** | 38±14.2% | 24±2.1% | 23±3.6% | 29±5.4% | 24±19.1% | 27±12.9% | 2±2.6% | 15±14.6% | 6±6.4% | 3±3.5% | 7±8.5% | 00±0% |

**Fig S3.** Inhibition percentages (IP, %) of *Aspergillus fumigatus* CBS 101355 in combinatorial application of and γAFPB6GXZ8 + fluconazole (FLC). Interaction ratio (IR) calculated according to the Abbot-formula presented below IP. Red cells indicate synergy (IR >1.5), orange cells additive interaction (IR between 0.5 and 1.5), while green cells antagonism (IR < 1.5). The untreated control was defined as 100% of growth.


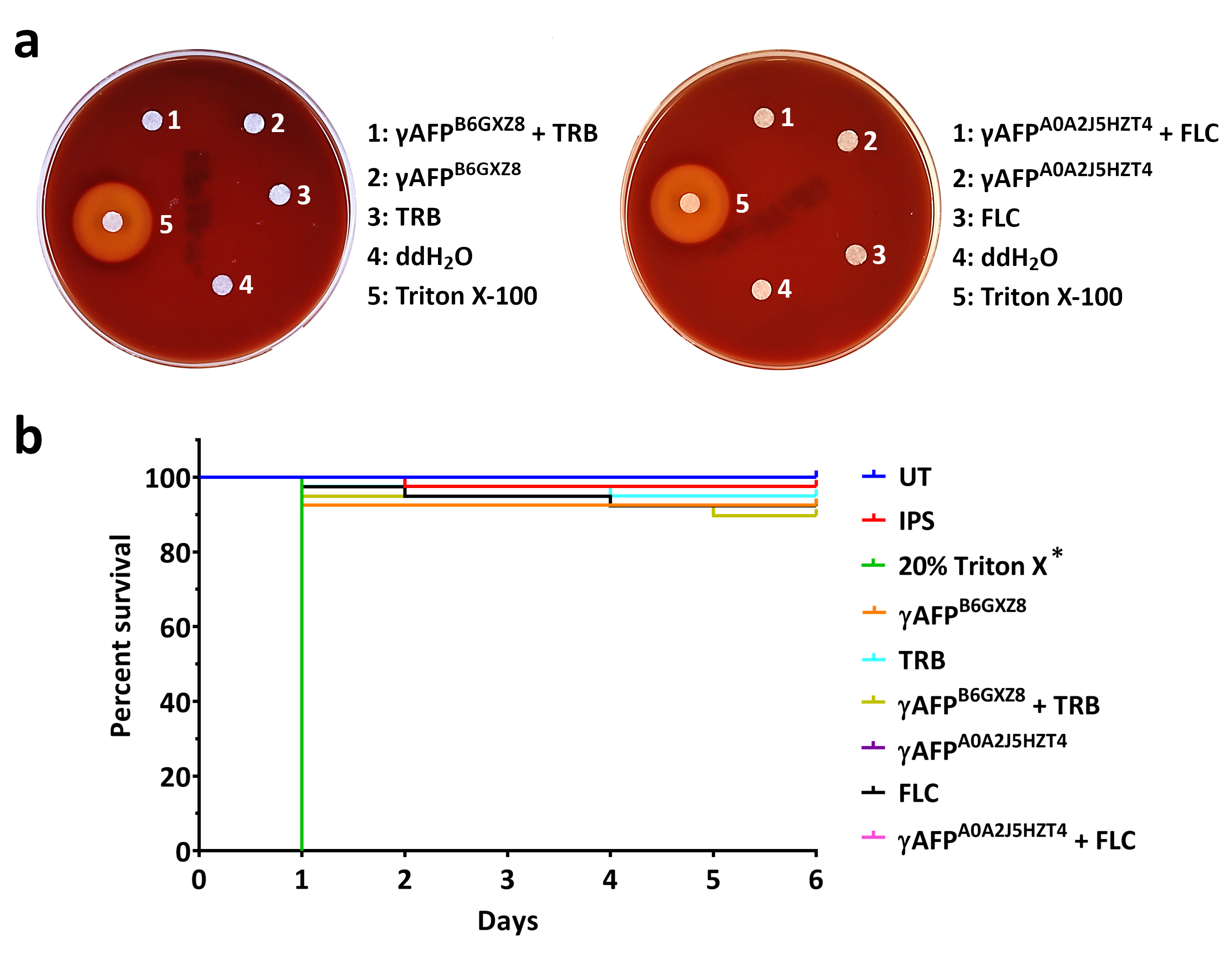


**Fig. S4.** Hemolytic activity of γAFP^B6GXZ8^ (200 µg/mL), γAFP^A0A2J5HZT4^ (200 µg/mL), TRB (1 µg/mL), FLC (32 µg/mL), and their respective synergistic combinations (γAFP^B6GXZ8^ [200 µg/mL] + TRB [0.5 µg/mL], γAFP^A0A2J5HZT4^ [200 µg/mL] + FLC [32 µg/mL]) on Columbia blood agar plates after incubation for 24 h at 37°C (**a**). Triton X-100 [20% (v/v)] and ddH2O were used as the positive and negative lysis controls, respectively. Sterile filter paper disks (diameter: 6 mm) were impregnated with 10 µL of each solution and placed onto agar plates. Survival of *Galleria mellonella* larvae after injection with 20 µL IPS solution of peptides, antifungal drugs, and their combinations in concentration that used in hemolytic activity test (**b**). UT: untreated control, IPS: insect physiological saline-treated control. *: p ≤ 0.05 from both Log rank (Mantel-Cox) and Gehan-Breslow-Wilcoxon tests.


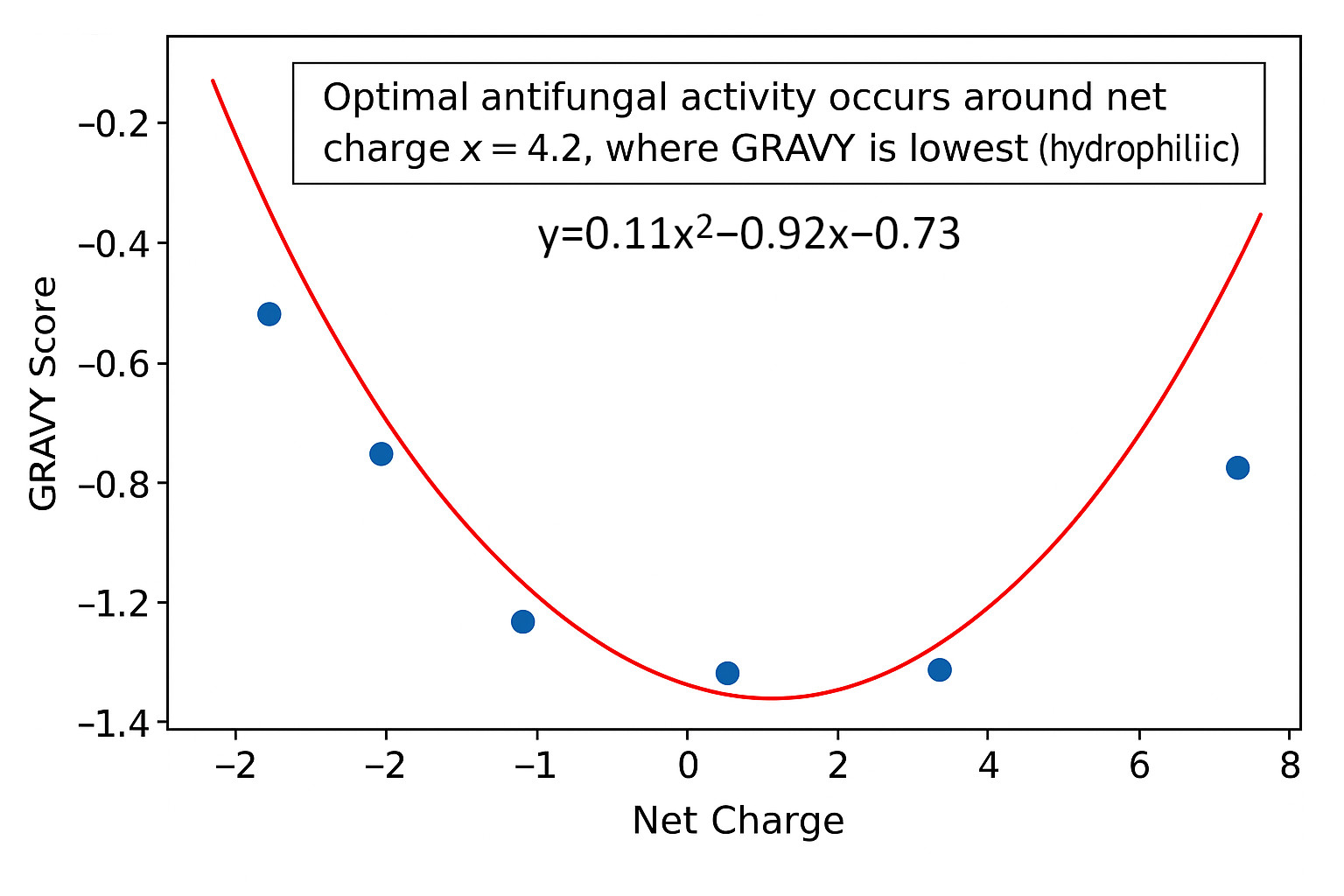


**Fig. S5.** Quadratic relationship between net charge and GRAVY score in antifungal active γAFPs. Blue dots represent antifungal active γAFPs highlighted in gray in Tables 1 and 2. The red curve shows a quadratic regression model.

The analysis revealed a non-linear relationship between net charge and GRAVY score. The fitted quadratic model y=0.11x^2^−0.92x−0.73 showed a minimum GRAVY value at net charge +4.2. This suggests that peptides with moderately positive net charge tend to be more hydrophilic, which may enhance their antifungal efficacy. The model achieved an R^2^ value of 0.65, indicating a moderate fit to the data.

Method: γAFPs with antifungal activity (Tables 1 and 2) were analyzed for net charge and GRAVY scores using standard bioinformatics tools. A quadratic regression model was fitted to the data using least squares estimation to explore the relationship between net charge and hydropathy. The model equation was derived and visualized using Python-based scientific plotting libraries.
